# Prototype Smartphone-Based Device for Flow Cytometry with Immunolabeling via Supra-nanoparticle Assemblies of Quantum Dots

**DOI:** 10.1021/acsmeasuresciau.1c00033

**Published:** 2021-11-05

**Authors:** Zhujun Xiao, Ghinwa H. Darwish, Kimihiro Susumu, Igor L. Medintz, W. Russ Algar

**Affiliations:** † Department of Chemistry, University of British Columbia, 2036 Main Mall, Vancouver, British Columbia V6T 1Z1, Canada; ‡ Jacobs Corporation, Hanover, Maryland 21076, United States; § Optical Sciences Division, Code 5600, U.S. Naval Research Laboratory, Washington, D.C. 20375, United States; ∥ Center for Bio/Molecular Science and Engineering, Code 6900, U.S. Naval Research Laboratory, Washington, D.C. 20375, United States

**Keywords:** immunofluorescence, flow cytometry, imaging, microfluidic, smartphone, quantum dots

## Abstract

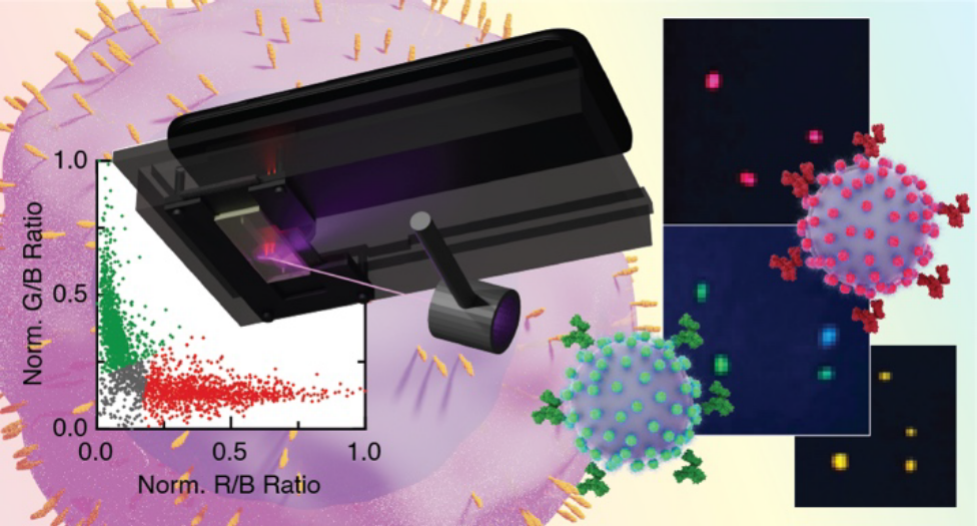

Methods for the detection, enumeration, and typing of cells are important in many areas of research and healthcare. In this context, flow cytometers are a widely used research and clinical tool but are also an example of a large and expensive instrument that is limited to specialized laboratories. Smartphones have been shown to have excellent potential to serve as portable and lower-cost platforms for analyses that would normally be done in a laboratory. Here, we developed a prototype smartphone-based flow cytometer (FC). This compact 3D-printed device incorporated a laser diode and a microfluidic flow cell and used the built-in camera of a smartphone to track immunofluorescently labeled cells in suspension and measure their color. This capability was enabled by high-brightness supra-nanoparticle assemblies of colloidal semiconductor quantum dots (SiO_2_@QDs) as well as a support vector machine (SVM) classification algorithm. The smartphone-based FC device detected and enumerated target cells against a background of other cells, simultaneously and selectively counted two different cell types in a mixture, and used multiple colors of SiO_2_@QD-antibody conjugates to screen for and identify a particular cell type. The potential limits of multicolor detection are discussed alongside ideas for further development. Our results suggest that innovations in materials and engineering should enable eventual smartphone-based FC assays for clinical applications.

## Introduction

Smartphones are emerging as a versatile platform for portable bioanalysis and imaging.^1−6^ The built-in light sources, cameras, batteries, network connectivity, and computing power of smartphones can enable or support many of the same capabilities as conventional benchtop spectroscopic and imaging instruments. Further considering their mass production, rapid advances in technology, and global ubiquity, smartphones are well-suited for applications requiring an analysis device with small size and relatively low cost. Modes of detection that utilize the smartphone camera have included optical density, colorimetry, and photoluminescence (PL), among others.^1−6^ Selected examples of smartphone-based analyses and imaging via PL include a diagnostic panel for iron and vitamin A deficiency,^7^ a lateral flow immunochromatographic assay for the detection of influenza A,^8^ direct analysis of enzyme activity in whole blood,^9^ detection of SARS-CoV-2 via real-time polymerase chain reaction (RT-PCR),^10^ detection of other respiratory pathogens via loop-mediated isothermal amplification (LAMP) of nucleic acids,^11^ an enzyme-free amplified assay for micro RNA,^12^ the detection of *Nosema ceranae* spores in honey bees,^100^ enumeration of an immunomagnetically isolated target cancer cell type,^13^ and many more.

Cellular analyses are an important subset of bioanalysis, with immunophenotyping by flow cytometry being one of the most widely used and powerful tools for this purpose.^14,15^ Contemporary flow cytometers (FCs) can measure double-digit numbers of fluorescently labeled biomarkers per cell and provide morphological information.^16−18^ Examples of applications include the detection of leukemias and lymphomas, immune-related disorders, circulating tumor cells, stem cells, fetal cells, and pathogenic bacteria.^19−23^ Unfortunately, FCs are often large and expensive instruments and often require operators with specialized training. They thus tend to be limited to well-funded research laboratories and shared core facilities and do not have the portability and low cost desired for point-of-need applications (e.g., field deployment, patient bedsides, points of primary care). Significant research efforts have therefore been directed toward small-scale platforms for cellular analysis based on, for example, microfluidics.^24−29^ Smartphones have good potential to add to these efforts.^30^

A general challenge with smartphones is that they have not been designed with scientific PL measurements in mind. Engineering of peripheral components and utilization of advanced and optimized photoluminescent materials are often necessary for adequate performance. With respect to the latter, we have shown that semiconductor quantum dots (QDs), semiconducting polymer dots, and supra-nanoparticle assemblies of QDs offer significant advantages over fluorescent dyes and fluorescent proteins.^31,32^ For QDs, in particular, their bright and spectrally narrow PL offers higher sensitivity and better multicolor capability with smartphone imaging.^31−33^

Here, we make an important advance toward a smartphone-based FC and immunophenotyping by developing a 3D-printed prototype and utilizing immunoconjugates of high-brightness SiO_2_@QD supra-nanoparticle assemblies. To date, smartphone-based immunofluorescent enumeration assays have generally captured target cells from a suspension sample.^13,34,35^ Imaging of static cells facilities adequately sensitive imaging on a smartphone but can have throughput limitations. Flow analyses on a smartphone have only utilized cells treated with fluorescent nuclear stains,^36,37^ where this abundant and nonspecific labeling provides sufficient brightness for measurements under flow, but at the cost of little or no molecular information. In contrast to both of the foregoing, our combination of a device and SiO_2_@QDs is capable of immunofluorescent analysis of cells in a flow stream, potentiating FC-like immunophenotyping on a smartphone. We show that our prototype smartphone-based FC device can detect a target cell type against a background of nontarget cells, concurrently and selectively enumerate two different cell types in a mixture, and screen for and identify a cell type based on the PL color of its immunolabeling. The overall capacity for multicolor detection is discussed alongside the strengths, weaknesses, and future improvements for the device. Altogether, our results suggest that continued materials development and device engineering should eventually enable smartphone-based FC assays with clinical relevance and utility.

## Results

### Device

**Figure 1:**
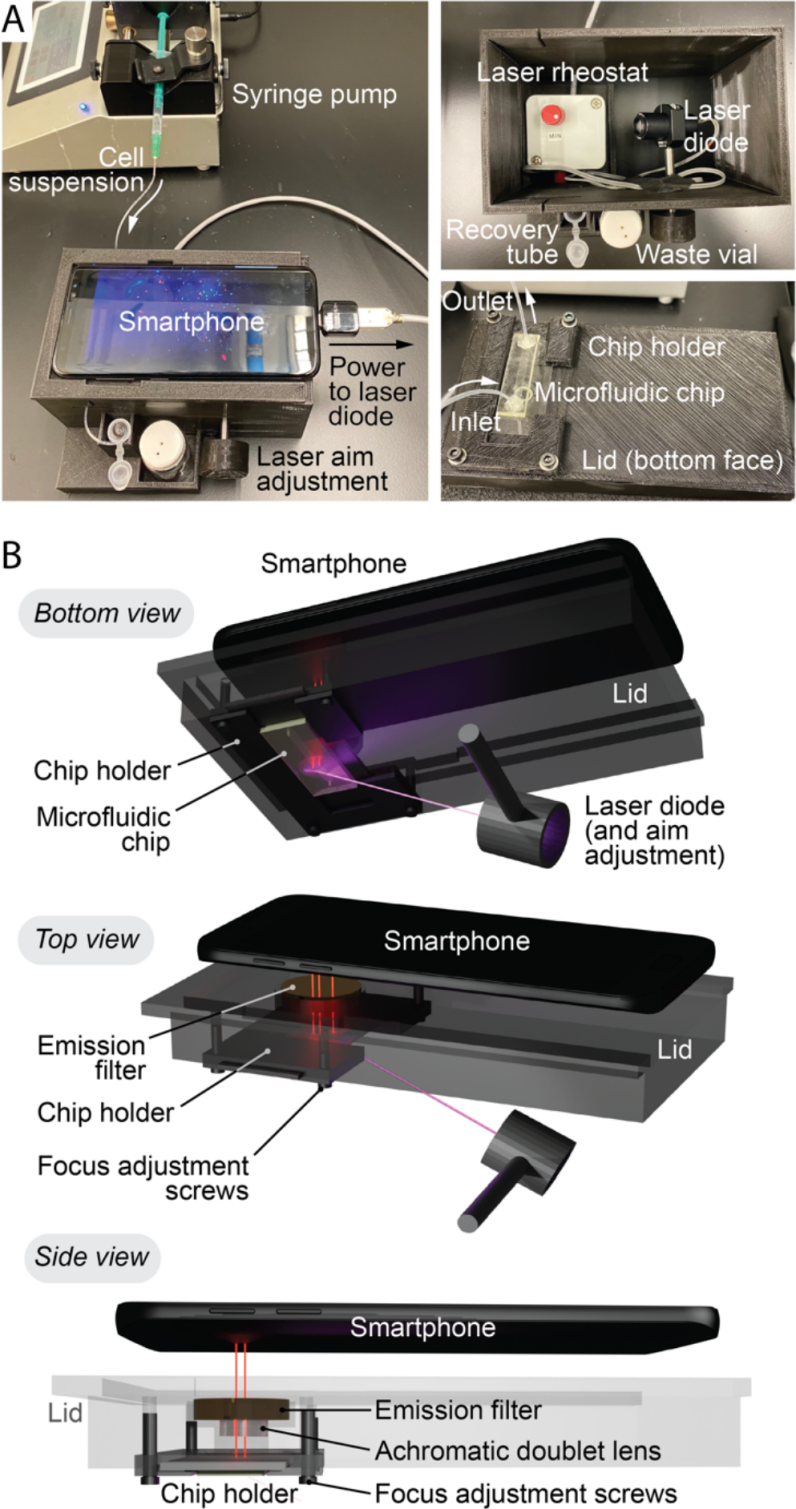
Design of the smartphone-based FC device. (A) Photographs of the 3D-printed device prototype. Video S1 is a short clip of the device in operation. (B) Renderings of the model of the prototype device from different viewpoints. The surrounding box is omitted for clarity. A schematic for the laser optics and a diagram and photograph of the microfluidic chip can be found in the SI.

Figure 1 illustrates the design of the 3D-printed smartphone-based FC. The smartphone stage was a lid for a dark box (length × maximum width × height = 15.1 cm × 12.3 cm × 9.3 cm, mass = 271 g) and held a 450 nm long-pass filter to block stray excitation light (vide infra) and an achromatic doublet lens to magnify the image. The chip holder aligned the channel of a PDMS-on-glass microfluidic chip with the magnified field-of-view (∼2.8 μm per pixel, 1920 × 1080 pixels = 5.4 × 3.0 mm) of the smartphone camera for imaging through the glass. To focus on the cells passing through the microfluidic channel, the distance between the doublet lens and the channel was adjustable via screws at all four corners of the chip holder. A laser diode (405 nm, max 20 mW) was the excitation source. It was powered by the smartphone, and its output intensity was adjustable via a rheostat. The laser output was shaped by lenses into a line profile (Figure S1) and was rotatable to different angles of incidence via an adjustment rod and knob. A sample (i.e., cell suspension) was introduced to the microfluidic chip (straight channel, ∼3 mm width, ∼100 μm height; Figure S2) through tubing connected to a syringe pump (typical flow rate of 10 μL/min). Effluent from the microfluidic chip was connected via additional tubing to either a waste vial or a sample recovery tube. Additional details and specifications for the components and design can be found in the Supporting Information (SI).

For measurements, the laser beam was directed to the section of the microfluidic chip within the smartphone camera field of view. With the laser switched on, the smartphone camera recorded video as cell suspension was flowed through the microfluidic chip. Video files were transferred to a personal computer and cropped to the area illuminated by the laser for analysis. The analysis algorithm tracked cells and analyzed their red-green-blue (RGB) color information for counting and classification. The size of the tracked objects was also factored into the cell counting algorithm to compensate for potential clusters of cells. Further information regarding the analysis can be found in the SI.

### Materials

**Figure 2:**
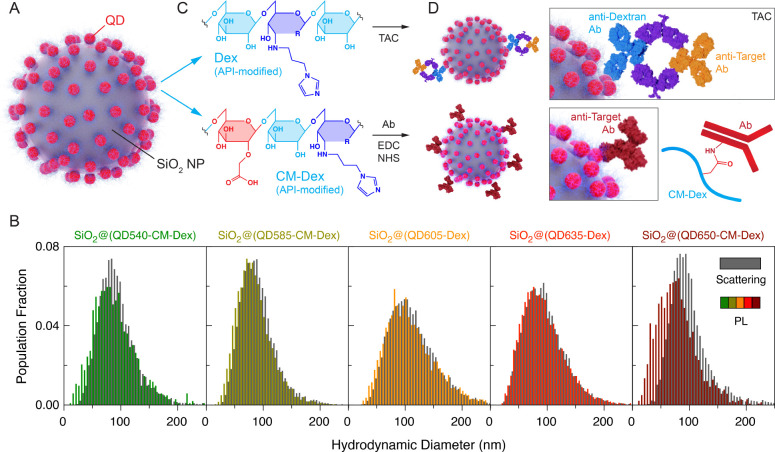
SiO_2_@QD immunoconjugates. (A) Diagram of a SiO_2_@QD and (B) representative examples of size distributions derived from NTA scattering mode and PL mode measurements. The QDs were further functionalized with (C) 1-(3-aminopropyl)imidazole (API)-modified dextran (Dex; top) or API-modified carboxymethyl dextran (CM-Dex; bottom). The structures are intended to be conceptually illustrative and do not reflect the actual stoichiometry or relative positions of the CM and API modifications along the dextran chain. (D) SiO_2_@(QD-Dex) were immunoconjugated via TACs (top), whereas SiO_2_@(QD-CM-Dex) were immunoconjugated via carbodiimide coupling (bottom).

We have previously reported the preparation and characterization of the SiO_2_@QD supra-nanoparticle assemblies, illustrated in Figure 2A.^38^ Nanoparticle tracking analysis (NTA) characterization of batches of SiO_2_@QD relevant to the present study are shown in Figure 2B. These assemblies had average diameters between 75–120 nm (see Table S1) and an estimated 50–60 QDs per SiO_2_ nanoparticle.^38^ Spectroscopic characterization can be found in the SI. For experiments that used a single color of SiO_2_@QD-antibody conjugate, this conjugation was done using tetrameric antibody complexes (TACs) in combination with a dextran (Dex) coating on the QDs (Figure 2C–D and Figure S5A), as reported previously.^38,39^ These conjugates are denoted as SiO_2_@(QDλ-Dex)-TAC, where λ is the peak PL emission wavelength of the QDs. Similar to our prior study,^38^ immunolabeling with SiO_2_@QD instead of single QDs offered significantly higher signal-to-background ratios and signal-to-noise ratios (Figure S6).

Although the TAC strategy is both facile and effective, it does not yet afford conjugation of specific antitarget antibodies to specific colors of QD. An alternate strategy was therefore required for the parallel use of multiple antibody conjugates. In these cases, immunoconjugates of SiO_2_@QD were prepared by carbodiimide coupling of antibodies to a carboxymethyldextran (CM-Dex) coating on the QDs (Figures 2C,D and S5B). These conjugates are denoted as SiO_2_@(QDλ-CM-Dex)-antibody.

Selective immunolabeling of multiple cell lines (SK-BR3, MCF-7, and MDA-MB-231 breast cancer cells, A549 lung cancer cells) with both SiO_2_@(QDλ-Dex)-TAC and SiO_2_@(QDλ-CM-Dex)-antibody conjugates was confirmed by fluorescence microscopy (Figures S7–S11). Antibodies were selected to target the SiO_2_@QDs to HER2 protein, CD44, mucin 1 (MUC1), epithelial cell adhesion molecule (EpCAM), and estrogen receptor (ER), although not all of these antigens were utilized in subsequent experiments.

### Counting a Single Cell Type

**Figure 3:**
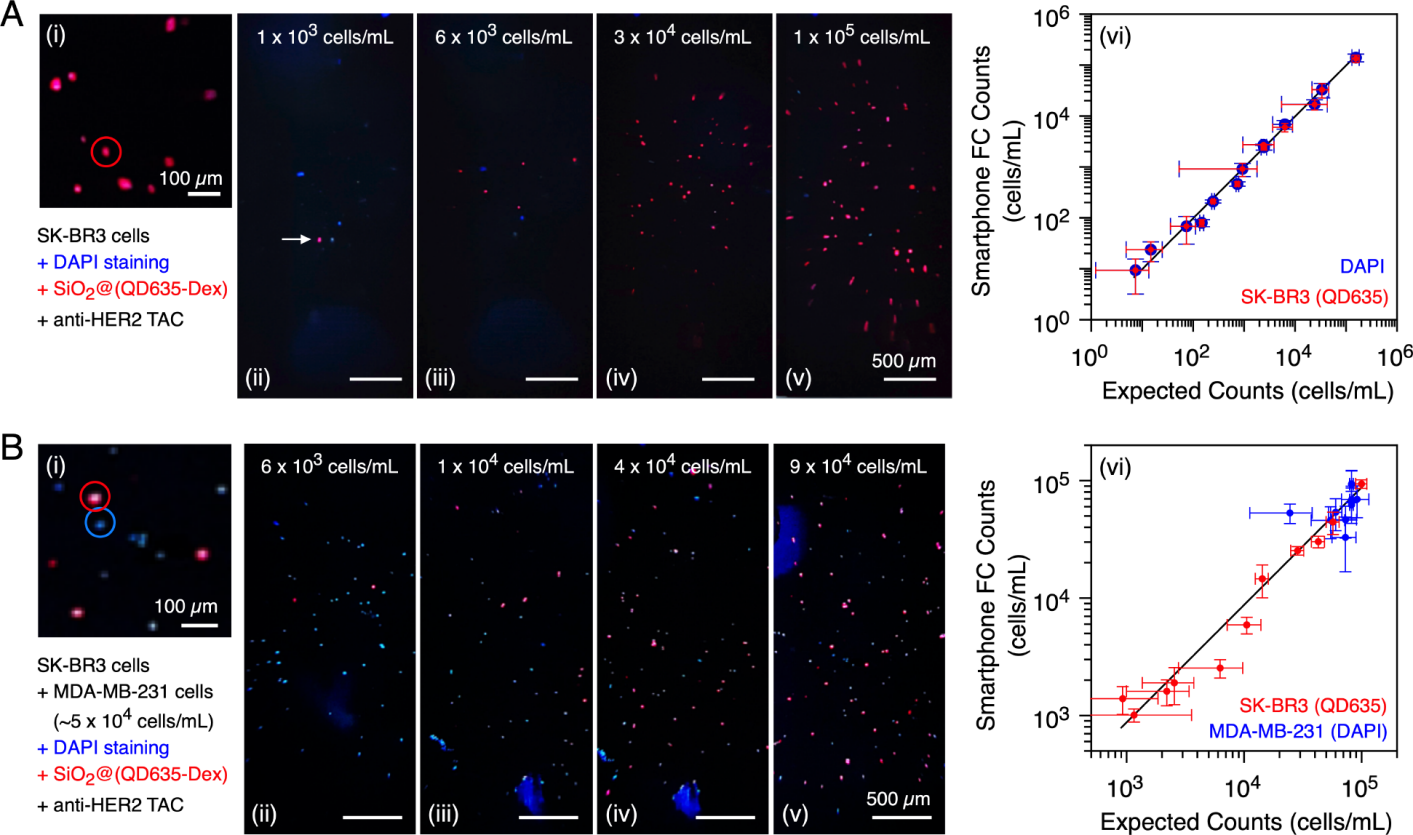
Counting of SK-BR3 cells, stained with DAPI (cell nuclei) and immunolabeled with SiO_2_@(QD635-Dex)-(anti-HER2 TAC). (A) SK-BR3 cells only: (i–v) frames from smartphone FC videos of different concentrations of suspended cells. For the most dilute suspension, the arrow indicates a single cell. An intensity profile across selected cells can be found in Figure S13. The image in (i) is a zoomed section of (v) with one (of several) SK-BR3 cells circled. Correlation plots (vi) of HER2-positive SK-BR3 cell counts derived from the smartphone FC versus a commercial cell counter. The diagonal line has a slope of unity. (B) Target SK-BR3 cells and background MDA-MB-231 cells: (i–v) frames from smartphone FC videos of mixed suspensions of SK-BR3 cells (variable amount) and MDA-MB-231 cells (approximately constant amount; DAPI stained). An intensity profile across selected cells of each type can be found in Figure S13. Video S3 is a 10 s clip from one of the smartphone FC videos. The image in (i) is a zoomed section of (v) with one example each of a SK-BR3 cell and a MDA-MB-231 cell circled. Correlation plots of cell counts (vi) measured with a smartphone FC versus a commercial cell counter. Frame image brightness has been digitally increased for display purposes.

Initial experiments were done with cells nonspecifically labeled with glutathione (GSH)-coated QD635, which bound efficiently to the membrane of ethanol-fixed SK-BR3 breast cancer cells. An approximately 1:1 correlation between cell counts was obtained for the smartphone FC versus a commercial cell counting instrument (see Figure S12). With this initial validation of device capability, subsequent experiments counted paraformaldehyde-fixed SK-BR3 cells that were specifically immunolabeled with SiO_2_@(QD635-Dex)-(anti-HER2 TAC). The HER2 (human epidermal growth factor receptor 2) gene and its downstream protein are overexpressed by SK-BR3 cells. The cell nuclei were also stained with DAPI dye. Representative frames from the smartphone FC videos are shown in Figure 3A(i–v). The immunolabeled SK-BR3 cells appeared red from the QD635 PL; however, the blue signal from DAPI staining was also present and revealed by a RGB color channel analysis. The 0.98(±0.02):1 correlation (i.e., slope) between the expected and measured smartphone FC cell counts from both the DAPI PL and QD635 PL are shown in Figure 3A(vi). The upper end of the dynamic range was estimated to be similar to 2 × 10^5^ cells/mL, being limited by the necessity for sufficient dark space between individual cells for reliable tracking. This suggested a maximum throughput on the order of 10^3^–10^4^ cells/min for flow rates between 10–50 μL/min. The lowest cell concentration that was measured was ∼10 cells/mL in a 200 μL volume with a 4 min sampling time.

Next, the counting experiment was repeated with mixtures of SK-BR3 cells and MDA-MB-231 cells, which do not express HER2. As shown in Figure 3B, the SK-BR3 cell counting was effectively unchanged by the presence of background HER2-negative cells, with no statistically significant difference in the correlation slope. Moreover, the HER2-negative MDA-MB-231 cells appeared blue from the DAPI staining and fluorescence and were clearly distinguishable from the red PL of the HER2-positive SK-BR3 cells. Counting of a specifically immunolabeled cell type against a count of total cells or MDA-MB-231 cells was thus possible.

### Multicolor Capability

**Figure 4:**
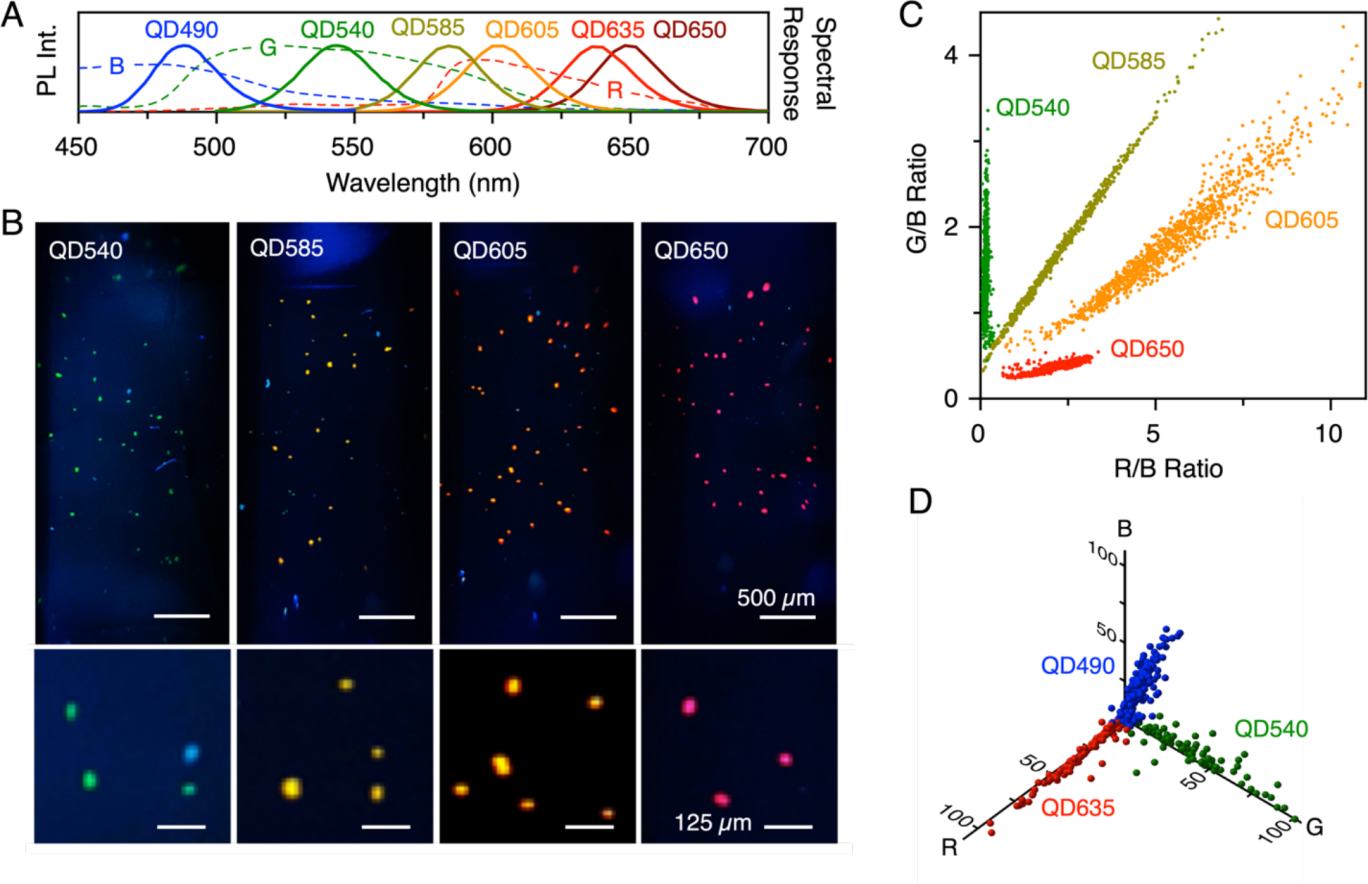
Assessment of multiple colors of QDs. (A) PL emission spectra of QD490, QD540, QD585, QD605, QD635, and QD650 (solid lines). The approximate RGB color filter transmission spectra for the smartphone are overlaid (dashed lines; data courtesy of Olivier Burggraaff^40^). (B) Frames from smartphone FC videos showing SK-BR3 cells immunolabeled with SiO_2_@(QDλ-CM-Dex)-(anti-HER2) for QD540, QD585, QD605, or QD650. Frame image brightness has been digitally increased for display purposes. Intensity profiles across selected cells of each color of QD can be found in Figure S16. (C) Plots of the G/B and R/B intensity ratios for individual SK-BR3 cells labeled with these four colors of SiO_2_@(QDλ-CM-Dex)-(anti-HER2). (D) Plots of the R, G, and B channel intensity values for individual SK-BR3 cells nonspecifically labeled with GSH-QD490, GSH-QD540, or GSH-QD635 (none as a SiO_2_@QD assembly).

Given the RGB color capabilities of the smartphone camera, we next evaluated the potential to distinguish multiple colors of the QD PL signal using the smartphone FC. In a first experiment, SiO_2_@(QD-Dex) were prepared using QD540 (green emission), QD585 (yellow emission), QD605 (orange emission), and QD635 (red emission). These materials were used, in separate experiments, to label fixed SK-BR3 cells via anti-HER2 TAC. PL emission spectra are shown in Figure 4A (along with spectra for QD490 and QD650, vide infra) with the transmission spectra for the RGB filters of the smartphone camera superimposed.^40^ Representative microscope PL images and smartphone FC video frames of immunolabeled cells are shown in Figure S14. In a second experiment, SK-BR3 cells labeled with four colors (QD540, QD585, QD605, QD650) of SiO_2_@(QDλ-CM-Dex)-(anti-HER2) were also imaged using the smartphone FC. Representative images are shown in Figure 4B. The video frames show visible differences in color, which can be quantified via the R and G channel intensities. Figure 4C is a plot of the R/B and G/B intensity ratios for the cells immunolabeled with each color of SiO_2_@(QD-CM-Dex)-(anti-HER2), where the B channel intensity served as a useful reference for improving precision. (The data without normalization to the B channel intensity can be found in Figure S15). The different colors of SiO_2_@QD clearly clustered along distinct lines that enabled unambiguous identification of each color. Notably, the green QD540 and red QD650 are approximately orthogonal to one another.

In the above context, blue-emitting QD490 was also tested alongside green-emitting QD540 and red-emitting QD635, albeit with nonspecific labeling of ethanol-fixed cells (Figure 4D). The color coordinate data shows the potential for three approximately orthogonal color channels. However, the blue QDs offered notably lower signal-to-background ratios than the other colors and were not used in subsequent experiments.

### Two-Plex Cell Counting

Given the capability to orthogonally resolve QD540 PL from QD650 PL, we evaluated the potential for parallel counting of two different cells, specifically SK-BR3 and MDA-MB-231 cells. The HER2 antigen (HER 2) and mucin-1 protein (MUC1) were used to target SK-BR3 cells and MDA-MB-231 cells, respectively, with SiO_2_@(QD540-CM-Dex)-(anti-HER2) and SiO_2_@(QD650-CM-Dex)-(anti-MUC1). As noted earlier, the prior Dex coating on the QDs in SiO_2_@QD assemblies was replaced with CM-Dex to enable carbodiimide coupling of antibodies. Prior to analysis, unlabeled cells and each type of SiO_2_@QD-labeled cell were separately run in the smartphone FC. The detection efficiency for unlabeled cells was much less than for immunolabeled cells, but some unlabeled cells were trackable (∼30%). The main contribution to the trackable signal was likely from scattered excitation light leaking through the long-pass filter of the device, although there may have been a minor contribution from weak cellular autofluorescence (which was detectable with a research-grade microscope). The G/B and R/B intensity ratio data from the smartphone FC videos were first normalized to values between 0 and 1 (see the SI for details) and used as a training set for a linear support vector machine (SVM) analysis model. Given the margins of separation between the different colors of SiO_2_@QD in the G/B versus R/B space in Figure 4C, SVM was an intuitive choice of classification algorithm. The SVM model classified tracked cells as green, red, or noncategorized (see the SI for details). The latter, determined from training with nonlabeled cells, corresponded to a region near the origin of G/B versus R/B plots (i.e., low signal levels in both the G and R color channels).

**Figure 5:**
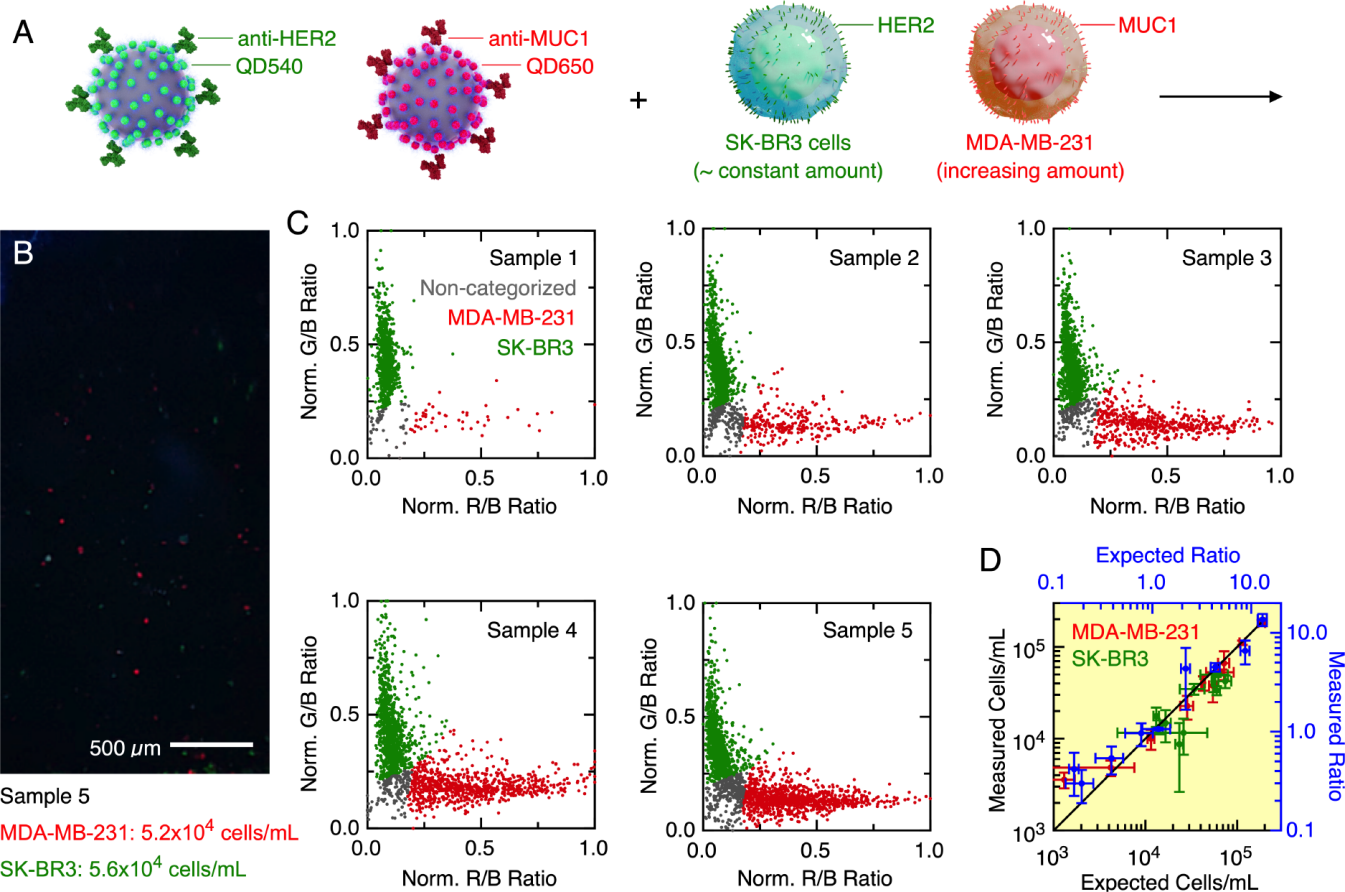
Counting of SK-BR3 and MDA-MB-231 cells in parallel using SiO_2_@(QD540-CM-Dex)-(anti-HER2) and SiO_2_@(QD650-CM-Dex)-(anti-MUC1), respectively. (A) Cartoon schematic of the experiment (not drawn to scale). (B) Example of a frame from a smartphone FC video. Frame image brightness has been digitally increased for display purposes. An intensity profile across selected cells of each type can be found in Figure S17. Video S4 is a 20 s clip from one of the smartphone FC videos. (C) Examples of normalized G/B and R/B intensity ratios and cell classifications for five samples with an increasing number of MDA-MB-231 cells and an approximately constant number of SK-BR3 cells (see Figure S18 for additional data sets). Normalization of intensity ratios assisted with application of the SVM training set to the experimental data (see the SI for details). (D) Correlation plots for the measured and expected numbers of SK-BR3 and MDA-MB-231 cells, and for the measured and expected ratios of MDA-MB-231 and SK-BR3 cells. This plot includes the samples in panel (C) and in Figure S18.

As shown in Figure 5A, SK-BR3 and MDA-MB-231 cells were mixed in suspension in different proportions and run through the smartphone FC. The samples had an approximately constant number of SK-BR3 cells and an increasing number of MDA-MB-231 cells. Figure 5B shows a representative frame from a smartphone FC video, and the cell counts, PL intensity ratios, and SVM classifications for five suspensions are shown in Figure 5C (see Figure S18 for analogous data for five different suspensions). Figure 5D shows the good correlations between the measured counts and expected counts for each cell line, and between the measured and expected ratios of the cell counts. Selective and concurrent enumeration of two cell types in a mixture was thus possible.

### Cell Identification

Differences in HER2, estrogen receptor (ER), and progesterone receptor (PR) expression levels are used, in part, to classify breast cancer cells as luminal A or B (ER+ and/or PR+, HER2– or HER2+), triple-negative/basal-like (HER2–, ER–, PR−), and HER2-enriched (HER2+, ER–, PR−), where this classification helps guide treatment.^41^ SK-BR3, MDA-MB-231, and MCF-7 breast cancer cell lines are examples of HER2 enriched, triple-negative, and luminal A types, respectively.^42^

**Figure 6:**
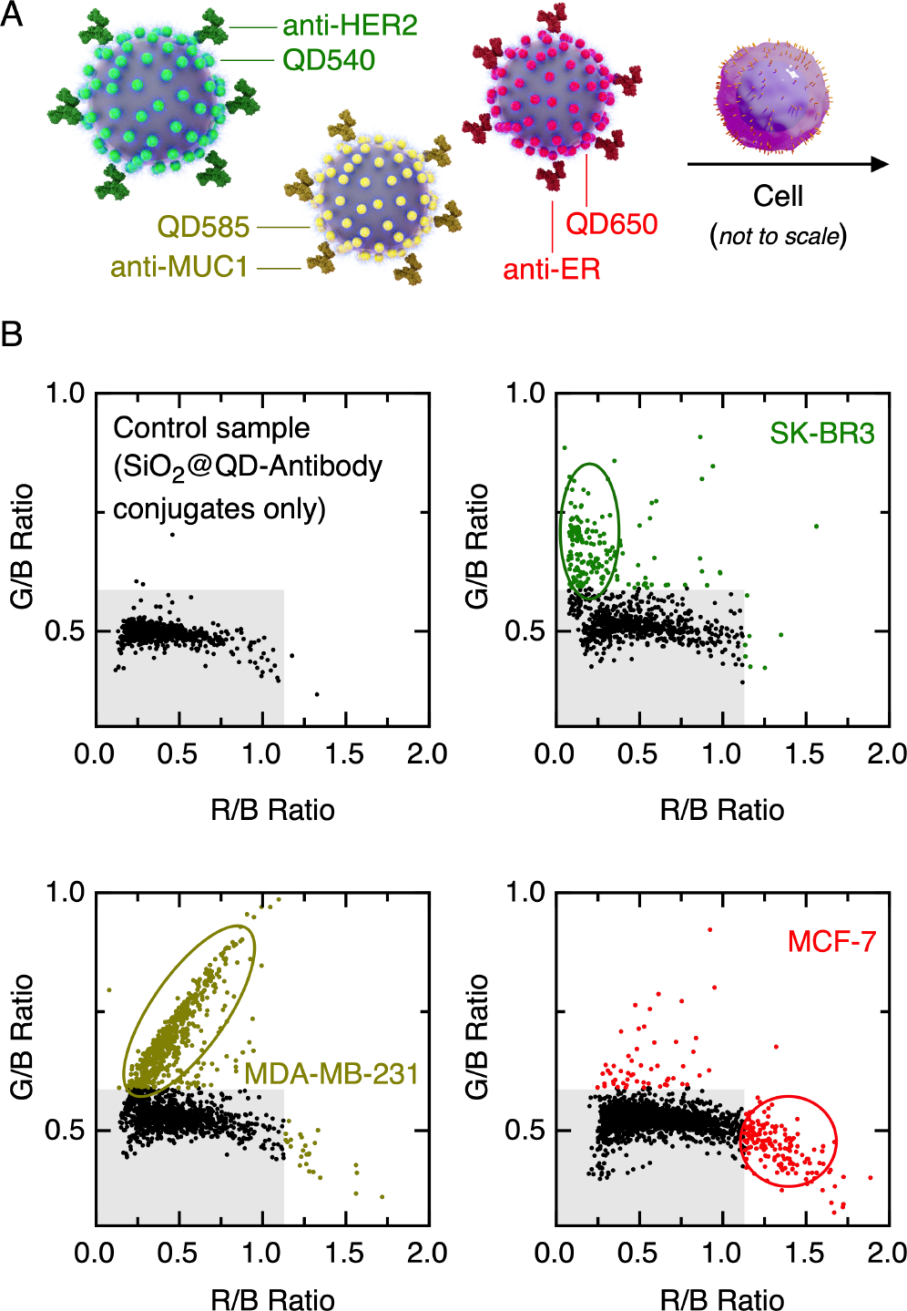
Identification of selected breast cancer cell types (SK-BR3, MDA-MB-231, and MCF-7) using SiO_2_@(QD540-CM-Dex)-(anti-HER2), SiO_2_@(QD585-CM-Dex)-(anti-MUC1), and SiO_2_@(QD650-CM-Dex)-(anti-ER). (A) Cartoon schematic of the experiment (not drawn to scale). (B) Examples of G/B and R/B intensity ratios for a control sample without cells and suspensions of each cell type. The shaded region in the plots corresponds to the mean of the control sample data points plus four standard deviations along each intensity ratio axis. The circled regions highlight the dominant vertical, diagonal, and horizontal spread of cell counts for SK-BR3, MDA-MB-231, and MCF-7 cells, respectively.

As an initial step toward the identification of breast cancer cell type with a smartphone FC, fixed SK-BR3 cells, MDA-MB-231 cells, or MCF-7 cells were incubated with SiO_2_@(QD650-CM-Dex)-(anti-ER), SiO_2_@(QD585-CM-Dex)-(anti-MUC1), and SiO_2_@(QD540-CM-Dex)-(anti-HER2) conjugates and then run through the smartphone FC (Figure 6A). The resulting plots of G/B intensity ratio versus R/B intensity ratio for single cell types are shown in Figure 6B. Notably, there was a recurring challenge with preparing SiO_2_@(QD650-CM-Dex)-(anti-ER) with robust colloidal stability, which was not observed with any other antibody conjugates. The presence of some aggregates of SiO_2_@QD650 was responsible for the cluster of objects detected in the absence of breast cancer cells and with all cancer cells. Although attempted, it was not possible to cleanly reject these aggregates from the analysis based on their size. Continued optimization of the SiO_2_@(QD650-CM-Dex)-(anti-ER) conjugates to avoid aggregates should improve the detection of the ER antigen and MCF-7 cells. Overall, the SK-BR3 cells gave a vertical profile (i.e., dominant QD540 labeling of HER2) on the G/B versus R/B plot of cell counts, MCF-7 cells gave a horizontal profile (i.e., dominant QD650 labeling of ER), and MDA-MB-231 cells gave a diagonal profile (i.e., dominant yellow labeling of MUC1). These trends were largely in agreement with expectations.^42^ The possible exception was the selectivity of labeling of MUC1, which might have been expected to be similarly expressed by both MCF-7 and MDA-MB-231 cells. One study has suggested that MUC1 expression follows the trend SK-BR3 < MDA-MB-231 ≤ MCF-7, albeit that there was significant variability between assay methods. In any case, discrimination between the breast cancer cells by the combination of anti-HER2, anti-MUC1, and anti-ER was possible, with the future potential for screening of breast cancer cell samples for classification as luminal (A or B), HER2 enriched, and triple-negative.

## Discussion and Conclusions

This work has shown strong potential for a smartphone-based FC. With sufficiently bright labels, such as SiO_2_@QD, immunolabeled extracellular markers associated with cells in a flow stream were detectable via video recording with a smartphone camera. Detection of DAPI nuclear staining was also possible and, in combination with immunolabeling, enabled counting of cell types of interest against a total cell count for the sample (Figure 3A). In the form of SiO_2_@QD, green-emitting QDs (QD540) and red-emitting QDs (QD635 or QD650) provided PL signals in the smartphone RGB color space that were effectively orthogonal to one another. This color pair is thus useful for the detection of two different cell types in mixture, provided that the two cell types have unshared markers that can be separately targeted with antibodies (Figure 3B). Indeed, our color data (Figure 4) suggests that up to four-plex detection should be possible in this context by using green-, yellow-, orange-, and red-emitting QDs and the smartphone RG color subspace. We also anticipate that a red-green color pair of QDs could be used for measuring the coexpression of two markers across a population of cells, and we plan to evaluate this capability in future work.

The distribution of PL intensities for each cell type and antigen was, in part, from variation in the antigen expression levels per cell. However, other likely contributions include some inhomogeneity in the excitation intensity across the microfluidic channel, variation in the vertical position of cells in the channel relative to the ideal focal plane, and variation in the linear velocities of cells. The quantitative impact of these instrumental factors will need to be assessed in future studies but was beyond the scope of this initial proof of concept. Moreover, the immunolabeling protocol was only optimized insofar as necessary to achieve labeling across a relatively wide range of cell concentrations. Further optimization may be possible for samples with known and approximately constant cell concentrations.

An immediate area for improvement of our prototype smartphone-based FC is optimization of the blue channel in the RGB color space. Although detection of DAPI staining was viable, signal-to-background ratios for immunolabeling with blue-emitting QDs were suboptimal. If the latter can be improved, the blue channel will add significant capability. A suitable blue-emitting QD will provide a third orthogonal color channel and measurement of the coexpression of three markers across a population of cells will become viable. For enumeration of cell types with distinct markers, different QDs may be able to access up to seven resolvable labeling colors (red, orange, yellow, green, blue, and two colors intermediate between green and blue; see Figure S19A). A double-digit number of resolvable labeling colors may be accessible through SiO_2_@QDs prepared with different mixtures of red-, green-, and blue-emitting QDs, as such mixed assemblies would be able to access the full RGB color space (Figure S19B). However, there are two important requirements for this hypothetical level of multiplexing: high labeling specificity (i.e., absolutely no shared expression of markers between cell types and no nonspecific binding); and high PL signal levels in order to avoid the convergence of the trajectories for all colors close to the (0,0,0) origin in the RGB color space (i.e., the lower the signals, the fewer the number of colors that can be distinguished).

A potential hardware improvement for the smartphone FC is the image magnification and resolution. At present, the imaging format provided some capacity to compensate for small aggregates of cells. Higher magnification and resolution, and reduced aberration (especially near edges of the field of view), may enable the morphological analysis of cells, similar to laboratory imaging flow cytometers. Other possible hardware improvements include replacing the conventional stand-alone syringe pump with a 3D-printed design that is integrated with the device dark box and powered by the smartphone or a portable power pack, and replacing the PDMS-on-glass microfluidic chip with a more robust transparent plastic chip. As the cell-counting detection limit (cells/mL) depends on the volume sampled, higher volumetric flow rates (mL/min) will enable lower limits of detection with shorter measurement times. However, the trade-off is that faster linear flow rates will result in shorter residence times in the smartphone camera field of view, and thus lower PL signal intensities. Faster volumetric flow rates would thus need to be compensated by a wider channel dimension and/or higher laser intensity in order for the PL signals from lesser-expressed cellular markers to remain detectable. A further technical improvement would be a custom smartphone app that allows cell classification and counting to be done on-smartphone instead of on a personal computer via the cloud.

In sum, we have demonstrated that smartphone-based immunofluorescent flow cytometry is possible with a relatively simple device design and high-brightness materials such as SiO_2_@QD. Our prototype smartphone FC offers far lower cost (≤$150 USD, plus the cost of the smartphone and syringe pump) and far greater portability than a conventional flow cytometer, albeit at the nontrivial cost of fewer detection channels, lower sensitivity, and other less favorable metrics of analytical performance. In the immediate future, smartphone FCs will not compete with conventional FCs as multipurpose laboratory research tools and clinical tools but do stand to be an advantageous platform for certain targeted applications. For example, with companion assay kits, smartphone-based FCs may become a diagnostic health care asset for communities in rural, remote, or less wealthy regions that lack access or cannot support a laboratory or clinical flow cytometry facility. Even in wealthy communities, smartphone FCs can be imagined to better enable and support personalized medicine at patient bedsides and also enable faster and more efficient diagnostic testing by primary care providers instead of by centralized clinical laboratories. Our work here is an important first proof of concept toward smartphone-based FC. It has demonstrated the potential for smartphone-based FC, shown how advanced luminescent materials are enabling, and identified opportunities and challenges for further development.

## Experimental Section

### Materials

CdSeS/ZnS QDs (QD540) were from CytoDiagnostics (Burlington, ON, Canada), CdSe/CdS/ZnS QDs (QD585, QD605, QD635, QD650) were synthesized using standard methods,^43,44^ and CdZnSe/Cd_0.2_Zn_0.8_S/ZnS QDs (QD490) were synthesized as described elsewhere.^45^ SiO_2_@QD were prepared as previously reported.^38^ API-modified dextran and CM-dextran were prepared using a published method,^13^ with further details available in the SI. Anti-HER2 antibody was from Novus Biologicals (Centennial, CO). Anti-EpCAM antibody, anti-CD44 antibody, anti-MUC1, and Do-It-Yourself Positive Selection Kit II were from STEMCELL Technologies (Vancouver, BC, Canada). Anti-ER antibody was from Abcam (Toronto, ON, Canada). A full list of reagents and antibody specifications can be found in the SI.

### Smartphone FC Device

The smartphone was a Samsung Galaxy S8. Videos were recorded with a frame rate of 30 fps with a duration of 2–4 min. The 3D-printed pieces (dark box, smartphone stage, sample holder, and laser diode mount) were designed with AutoCAD 2018 AutoDesk Student 3D drafting software (AutoDesk, San Rafael, CA) and printed out using a fused deposition modeling (FDM)-type 3D printer (Ender-3 Pro, Creality, Shenzhen, China) with black polylactic acid (PLA) filament. The laser diode (405 nm, 20 mW) was controlled by a circuit reported previously.^13^ The optical components in the device lid were a 450 nm cutoff long-pass filter (Thorlabs, Newton, NJ) and an achromatic doublet lens (10 mm focal length, 6 mm diameter, Thorlabs). The PDMS microfluidic chip was prepared by soft lithography, where the mold for the chip was fabricated by digital light processing (DLP)-type 3D-printing (Miicraft+ or Miicraft 50, MiiCraft & Creative CADworks, Toronto, ON, Canada). Full details can be found in the SI.

### Cytometry Experiments

SK-BR3, MDA-MB-231, MCF-7, and A549 cells were cultured using standard protocols, processed as a suspension in buffer, and fixed with paraformaldehyde. For experiments with TAC, fixed cells were mixed with preformed TAC complexes for 15 min in phosphate-buffered saline (PBS, pH 7.4) prior to adding SiO_2_@(QD-Dex) for an additional 15 min. The labeled cells were washed and resuspended in PBS buffer. For experiments with SiO_2_@(QD-CM-Dex)-antibody conjugates, the desired antibodies were coupled to the desired color of SiO_2_@QD in a one-pot reaction with 1-ethyl-3-(3-(dimethylamino)propyl)carbodiimide and *N*-hydroxysuccinimide. Excess reagents and uncoupled antibodies were removed by centrifugation and washes with buffer. Fixed cells were mixed with purified SiO_2_@(QD-CM-Dex)-antibody conjugates in Easy Sep buffer for 1 h and then washed and resuspended in PBS buffer. Additional details of these methods can be found in the SI.

Immunolabeled cell samples were injected into the microfluidic chip via a syringe pump, and smartphone videos (typically 2–3 min, 20–30 μL of cell suspension) were recorded. The flow rate was usually 10 μL/min, but was increased to 50 μL/min for low cell concentrations. The video files were transferred to a computer for analysis with a MATLAB algorithm. This algorithm identified and tracked cells as objects and determined their areas and mean R, G, and B channel intensities. A SVM model was trained with cells labeled with a known color of SiO_2_@QD, and this model was subsequently applied to the classification of cells in mixed suspension samples. Full details can be found in the SI.

## Supporting Information Available

Experimental methods and results, including materials and microfluidic chip preparation, microscopy and smartphone FC optics details, cell culturing, immunolabeling, and assay procedures, details of smartphone video analysis and cell classification, NTA characterization of selected SiO_2_@QD materials, additional results for cell immunolabeling and microscopy or smartphone FC results, illustrations of potential RGB multiplexing levels (PDF)
Video showing the smartphone-based device in operation (MP4)
Video of ethanol-fixed SK-BR3 cells nonspecifically labeled with GSH-QD635 (MP4)
Video of SK-BR3 cells stained with DAPI and immunolabeled with SiO_2_@(QD635- Dex)-(anti-HER2 TAC) in the presence of DAPI-stained MDA-MB-231 cells (MP4)
Video of SK-BR3 and MDA-MB-231 cells in parallel using SiO_2_@(QD540-CM-Dex)-(anti- HER2) and SiO_2_@(QD650-CM-Dex)-(anti-MUC1), respectively (MP4)
