# Supplementary material for: Prototype Smartphone-Based Device for Flow Cytometry with Immunolabeling via Supra-nanoparticle Assemblies of Quantum Dots
Source: ACS Meas Sci Au. 2021 Nov 5;2(1):57–66. doi: 10.1021/acsmeasuresciau.1c00033 (PMC9838726; doi:10.1021/acsmeasuresciau.1c00033)
Supplement: Supplementary file 1 — tg1c00033_si_001.pdf [file tg1c00033_si_001.pdf]

SUPPORTING INFORMATION

Prototype Smartphone-Based Device for Flow Cytometry with Immunolabeling via Supra-nanoparticle Assemblies of Quantum Dots

*Zhujun Xiao,[1] Ghinwa H. Darwish, [1] Kimihiro Susumu,[2,3] Igor L. Medintz,[4] W. Russ Algar[1]**

[1] Department of Chemistry, University of British Columbia, 2036 Main Mall, Vancouver, British Columbia, V6T 1Z1, Canada

[2] Jacobs Corporation, Hanover, Maryland 21076, United States

[3] Optical Sciences Division, Code 5600, U.S. Naval Research Laboratory, Washington, DC 20375, USA

[4] Center for Bio/Molecular Science and Engineering, Code 6900, U.S. Naval Research Laboratory, Washington, DC 20375, USA

*Corresponding author: algar@chem.ubc.ca

Table of Contents

Supplemental Experimental Section S-4

Materials...S-4

Microscopy..S-4

Smartphone FC Laser Optics...S-5

Microfluidic Chip Preparation..S-6

Preparation of GSH-QDs S-8

Preparation of His-QDs..S-8

Preparation of SiO_2@QD Assemblies S-8

Cell Culture S-10

Cell Fixation S-10

Smartphone FC Counting of Non-Specifically Labeled Cells (Figure S12)..........................S-11

Formation of Tetrameric Antibody Complexes (TACs)...S-12

Labeling of Fixed SK-BR3 Cells with Dex-QDs and SiO_2@(QD-Dex) via TACs (Figure S6, Figure S7, Figure S14)...S-12

Immunoconjugation of SiO_2@(QD-CM-Dex) S-12

Smartphone FC Counting SK-BR3 Cells Labeled with SiO_2@(QD635-Dex)-(anti-HER2 TAC) in the Absence of Background MDA-MB-231 Cells (Figure 3A) S-13

Smartphone FC Counting SK-BR3 Cells Labeled with SiO_2@(QD635-Dex)-(anti-HER2 TAC) in the Presence of Background MDA-MB-231 Cells (Figure 3B)................................S-14

Selective Cell Labeling and Counting with SiO_2@(QD-CM-Dex)-Antibody Conjugates......S-15

Smartphone FC Classification of Breast Cancer Cell Lines with SiO_2@(QD-CM-Dex)-antibody conjugates (Figure 6 and Figure S11)...S-16

Cell Tracking and Counting Algorithm S-17

Support Vector Machine (SVM) Classification S-19

Supplemental Results and Discussion S-21

Characterization of SiO_2@QD Assemblies...S-21

Immunolabeling Strategies...S-22

Comparison of Immunolabeling with QDs versus SiO_2@QD S-22

TAC Immunolabeling of Various Antigens on Cancer Cells..S-23
Verification of Selectivity of Carbodiimide-based Immunoconjugation...............................S-25
Immunolabeling of Different Antigens with SiO_2@(QD-CM-Dex) S-26
Counting Non-Specifically Labeled Cells...S-29
Counting a Single Cell Type S-30
Multicolor Immunolabeling S-31
Additional Data for Two-Plex Cell Counting S-34
Potential Multiplexing Levels S-36

Supplemental References...S-37

Supplemental Experimental Section

Materials. Anti-HER2 antibody (Cat. No. NBP2-32863, Clone HRB2/282, IgG1 Kappa) was from Novus Biologicals (Centennial, CO). Anti-EpCAM antibody (Cat. No.60147, Clone 5E11.3.1, IgG1 Kappa), anti-CD44 antibody (Cat. No. 60068, Clone IM7, IgG2b Kappa), anti-MUC1 (Cat. No. 60155, Clone 16A, IgG1 Lambda), and Do-It-Yourself Positive Selection Kit II (Cat. No. 17698) were from STEMCELL Technologies (Vancouver, BC, Canada). Anti-ER antibody (Cat. No. ab66102, C-542, IgG1) was from Abcam (Toronto, ON, Canada).

Dextran (*Leuconostoc mesenteroides*, 9000–11 000 Da or *Leuconostoc* spp. ~6000 Da) and carboxymethyl dextran (CM-Dex, *Leuconostoc mesenteroides*, 9000–11 000 Da), sodium (meta)periodate ($NaIO_4$), 1-(3-aminopropyl)imidazole (API), bovine serum albumin (BSA), triethoxy-3-(2-imidazolin-1-yl)propylsilane (>97%, IPS), trichloro(1H,1H,2H,2H-perfluorooctyl)-silane (PFOTS), pluronic F127, sodium cyanoborohydride ($NaCNBH_3$), tetramethyl ammonium hydroxide (TMAH), L-histidine (≥99%), and reduced L-glutathione (GSH) were from Sigma-Aldrich (Oakville, ON, Canada). Ammonia solution (28–30% w/w) was from VWR (Mississauga, ON, Canada). Sodium tetraborate decahydrate was from Amresco (Solon, OH).

CdSeS/ZnS QDs (QD540) were from CytoDiagnostics (Burlington, ON, Canada), CdSe/CdS/ZnS QDs (QD585, QD605, QD635, QD650) were synthesized using standard methods, and $CdZnSe/Cd_{0.2}Zn_{0.8}S/ZnS$ QDs (QD490) were synthesized as described previously.[1]

Deionized water was from a Milli-Q Synthesis water purification system (Millipore, Burlington, MA). 1X PBS buffer was from Gibco Life Technologies. The composition of this buffer was pH 7.4, 1.05 mM KH_2PO_4, 155 mM NaCl, 2.97 mM Na_2HPO_4, without calcium and magnesium ions. Easy Sep buffer was the above PBS buffer supplemented with 2% v/v fetal bovine serum (Sigma Aldrich) and 1 mM EDTA (Fisher Scientific, Ottawa, ON, Canada).

Microscopy. Cell images were acquired with an IX83 inverted epifluorescence microscope (Olympus, Richmond Hill, ON, Canada). This microscope was equipped with an X-Cite 120XL

metal-halide light source (Excelitas Technologies, Mississauga, ON, Canada), a white-light LED transmitted light source, an Orca-Flash 4.0 V2 sCMOS camera (C11440; Hamamatsu Photonics, Hamamatsu, SZK, Japan), motorized filter wheels (Sutter Instruments, Novato, CA), and MetaMorph/MetaFluor software (Molecular Devices, Sunnyvale, CA). Images were analyzed with Image J software. Excitation, dichroic, and emission filters were from Chroma Technology (Bellows Falls, VT). Objective lenses (Olympus) were 4X/0.16NA, 10X/0.4NA, and 60X/0.9NA. The microscope also had optics for differential interference contrast (DIC) imaging.

PL emission spectra for cells labeled with SiO_2@(QD-Dex) were acquired with a CCD spectrometer (Greenwave 16 VIS-50; StellarNet, Tampa, FL) that was coupled to the trinocular head of the microscope via a fiber-optic cable. The spectra were obtained with SpectraWiz software (StellarNet). A 530 nm long-pass filter was used to block the excitation light.

Smartphone FC Laser Optics. Figure S1 shows a diagram of the optics used to form the laser beam into a line-like profile for transverse illumination across the width of the microfluidic channel. The laser profile was 3 mm (length; across channel) × 2 mm (width). The output from the laser diode (D405-20; 405 nm, 20 mW, 5 V, 75 mA, Radial, Can, 3 Lead, 5.6 mm, TO-18; US-Lasers, Baldwin Park, CA, USA) was passed through an aspheric lens (f =3.3 mm) and a cylindrical lens (f =12.5 mm) to obtain the line-like profile. The laser diode and lenses were housed in an anodized aluminum lens tube (Thorlabs, Newton, NJ).

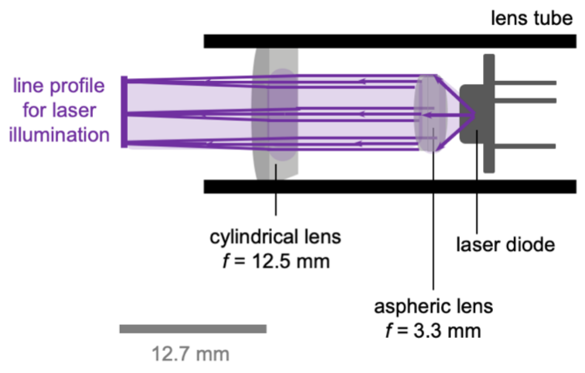

Figure S1. Optical schematic for obtaining a line-like beam profile from the laser diode in the smartphone FC device. The laser was aimed so that the line profile crossed the width of the microfluidic channel.

Microfluidic Chip Preparation. The microfluidic chip mold was designed on AutoCAD 2018 (AutoDesk, San Rafael, CA, USA) with a channel dimension of 28.8 mm × 3 mm × 0.1 mm (length × width × height), as shown in Figure S2A-B.

First-generation molds were printed out with a MiiCraft+ 3D printer (MiiCraft & Creative CADworks, Toronto, ON, Canada) and clear BV007A Microfluidics resin (Young Optics Inc., Taiwan). The width of the channel was set as the maximum dimension that was still fully visible under the smartphone camera with the magnification lens. The height of the feature for the channel was set based on the minimum z-resolution of the 3D printer. With a smaller channel height, cells tended to flow within the focal plane, which was beneficial for video analysis. After the mold was printed, it was rinsed with isopropanol (IPA) to remove excess resin, dried with compressed air, and exposed to UV light for 4 h for curing and further strengthening. The mold was then washed with a series of alcohols (ethanol, methanol, and IPA in order), followed by a heating process at 70 °C in an oven for 4 h to remove any residual resin on the mold surface that might cause incomplete curing of the polydimethylsiloxane (PDMS).

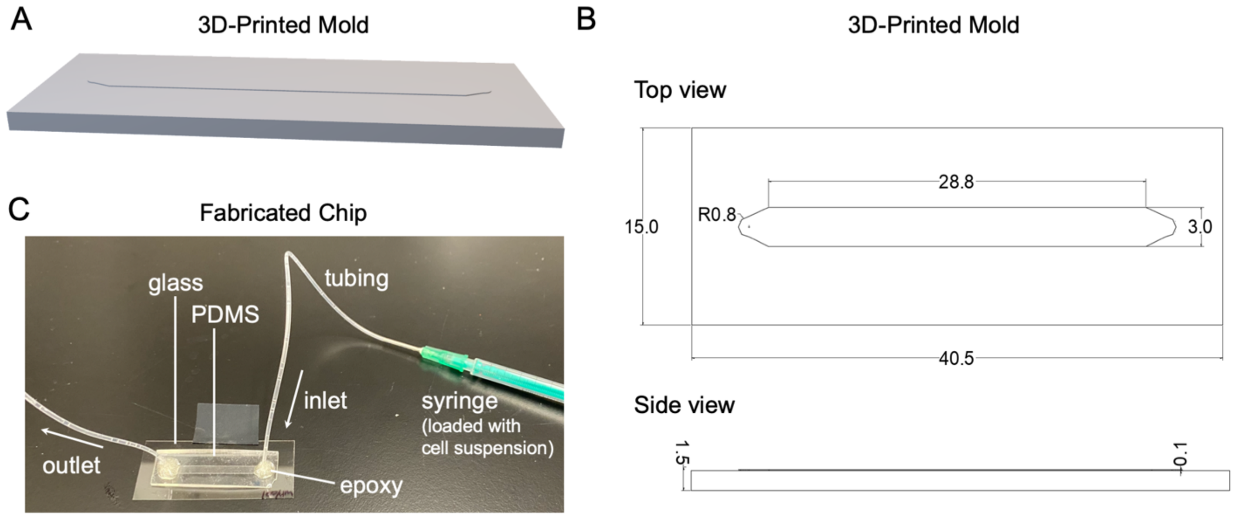

Figure S2. Microfluidic chip design. **(A)** Rendering of the 3D-printed mold for casting PDMS microfluidic channels. **(B)** Dimensions of the 3D-printed mold. The units are millimeters. **(C)** Photograph of a fabricated PDMS-on-glass microfluidic chip, connected with tubing, for use in the smartphone FC device.

Second-generation molds for microfluidic chips were printed with a MiiCraft 50 3D printer (MiiCraft & Creative CADworks, Toronto, ON, Canada) and resin specifically designed for use with PDMS soft lithography (Master Mold for PDMS, ResinWorks3D, Toronto, ON, Canada). This resin does not require post-printing heating of the mold.

For both generations of mold, the surface was activated using an air plasma cleaner at its high RF level (18W, Harrick Plasma PDC-32G; Ithaca, NY, USA) for 2 min. The mold surface was reacted with PFOTS under vacuum in a desiccator for 30 min to create a hydrophobic surface. This post-treatment of the mold was important for satisfactory PDMS casting results.

PDMS microfluidic chips were prepared by mixing the monomer and the curing agent together at a 10:1 ratio by mass, followed by degassing under vacuum until there were no bubbles. After the mixture turned clear, it was poured onto the mold and cured overnight in an oven at 70 °C. The PDMS chip was then peeled from the mold and cut to the desired size. Holes with a radius of ~0.8 mm were created with a metal punch at each end of the channel to be the inlet and outlet. Glass coverslips and PDMS chips were put in the air plasma cleaner for 2 min at the high RF level and then adhered to each other immediately after the plasma oxidization. As shown in Figure S2C, polyethylene tubing (30 cm long, 0.86 mm ID/1.27 mm OD) was attached to the inlet and outlet holes of the PDMS chip and sealed with epoxy (5 Minute Epoxy; Devcon Ltd., Danvers, MA). The other end of the inlet tubing was attached to a 1 mL syringe with a blunt needle (21G, 0.80 mm OD) for sample injection.

Before the first measurement with a cell suspension, the microfluidic chip was treated in the air plasma cleaner for 1 min. Next, 0.08% v/v pluronic F127 (aq) was then flowed through the channel at a speed of 10 µL/min for 30 min followed by a 0.5% w/v BSA solution in 1X PBS buffer at 10 µL/min for 30 min to make the surface hydrophilic and reduce cell adhesion to the channel walls during measurements. The PDMS treatment process was repeated if the microfluidic chip was still in use after 24 h. We found that a chip could be reused until a large number of cells started to stick to the PDMS channel, even after re-treatment. Typically, a chip was reusable for 20–30 runs, with a higher number of re-uses possible for lower cell concentrations in samples than higher cell concentrations.

Preparation of GSH-QDs. GSH-QDs were prepared by dissolving 80–100 mg of GSH in 300 µL of methanolic tetramethylammonium hydroxide (TMAH; 25% w/w in methanol). Hydrophobic QDs (~81 µM, 20 µL) were diluted with 900 µL of chloroform in a 1.7 mL microcentrifuge tube. The GSH solution was then added to the hydrophobic QDs and mixed by vortex. The mixture was kept at room temperature in the dark for 8–12 h. Borate buffer (200 µL, 50 mM, pH 9.2) was then added to extract the GSH-QDs. After the organic and aqueous phases separated, the GSH-QDs were collected with the aqueous phase and the organic phase was discarded. Ethanol (~800 µL) was then added to precipitate the aqueous GSH-QDs, which were pelleted by centrifugation at 4800 RCF for 10 min. The supernatant was discarded to remove free ligands and excess TMAH. The GSH-QDs were then redispersed in borate buffer (100 µL, 50 mM, pH 9.2). This ethanol precipitation process was repeated another two times before the GSH-QDs were finally redispersed in borate buffer (200 µL, 50 mM, pH 9.2). The GSH-QDs were stored at 4 °C until needed.

Preparation of His-QDs. Histidine-coated QDs (His-QDs) were prepared by first diluting hydrophobic QDs (~81 μM, 10 µL) in chloroform (900 µL). L-histidine (0.645 mmol, 100 mg) was dissolved in TMAH (300 µL, 25% w/w in methanol) and added to the QD solution. The mixed sample was kept in the dark at room temperature for 1 h. Borate buffer (200 µL, 50 mM, pH 9.2) was then added to extract the His-QDs. After the organic and aqueous phases separated, the His-QDs were collected with the aqueous phase and the organic phase was discarded. His-QDs were collected via centrifugation at 4800 RCF for 10 min and redispersed in borate buffer (100 µL, 50 mM, pH 9.2) and precipitated with ethanol (~800 µL) to remove free ligands. After three wash cycles, the final pellet was redispersed in borate buffer (100 µL, 50 mM, pH 9.2) and stored at 4 °C until needed.

Preparation of SiO_2@QD Assemblies. SiO_2@QD self-assemblies were prepared using a previously published method.[2] In brief, SiO_2 nanoparticles (SiO_2 NPs) were synthesized with a modified Stöber method[3] and then functionalized with triethoxy-3-(2-imidazolin-1-yl)propylsilane (IPS). SiO_2 NPs in ethanol (100 µL) were mixed with IPS (10 µL, 50% v/v IPS in ethanol) and NH_3OH (10 µL, aq, 28–30% w/w) at room temperature on a shaker for 2.5 h. IPS-modified SiO_2 NPs (SiO_2@IPS) were collected via centrifugation at 8400 RCF for 10 min and redispersed in borate buffer (100 µL, 50 mM, pH 8.5). Three cycles of precipitation and redispersion were used

to wash the SiO_2@IPS. Next, the SiO_2@IPS (50 µL) were then mixed with GSH-QDs (12 µL, ~3 µM) for 1 h at room temperature in the dark. SiO_2@QD self-assemblies were then collected via centrifugation at 10 000 RCF for 10 min, followed by three cycles of centrifugation and redispersion in borate buffer (100 µL, 50 mM, pH 8.5).

(3-Aminopropyl)-imidazole (API)-modified dextran[4] was used to functionalize both single QD635 and SiO_2@QD635 for cell labeling:

Dextran (0.50 g, 9–11 kDa, *Leuconostoc mesenteroides*) was dissolved in 20 mL of deionized water in a 40 mL glass vial. $NaIO_4$ (3.1 mL, 0.1 M (aq)) was added to the dextran solution. The mixture was covered with aluminum foil and stirred overnight at 4 °C in the dark. The samples were then purified by dialysis against 1.0 L of deionized water using 3.5 kDa-MWCO dialysis tubing for 2 days with three water changes. The purified sample was freeze-dried and the final product was a fluffy white solid (Ox-Dex). The Ox-Dex (0.1 g) was dissolved in 2 mL of deionized water in a 20 mL glass vial. API (0.25 mL, 51 mg/mL in deionized water) was added and the reaction was mixed overnight at room temperature. An aliquot of $NaCNBH_3$ (0.25 mL, 51 mg/mL) was added to the solution and left to mix overnight at room temperature. The sample was then pipetted into ethanol/hexane (95%/5% v/v) solution to induce precipitation and pelleted via centrifugation at 13 000 RCF for 5 min. The supernatant was discarded, and the pellet was redissolved in 2 mL of deionized water. The precipitation with ethanol/hexane was repeated and the final product (API-Dex) was dried under reduced pressure. A procedure analogous to the foregoing was used for preparing API-modified carboxymethyl dextran (CM-Dex-API, 9–11 kDa), and for different colors of SiO_2@QD.

To prepare API-Dex-functionalized single QD635 (QD635-Dex), 15 mg of API-Dex was first dissolved in carbonate buffer (250 µL, 100 mM, pH 9.3). His-QD635 (20 µL, 8.3 µM) was added to the solution and mixed via vortex. This solution was incubated at 60 °C for 3 h in the dark. Dex-QD635 were purified via three rounds of centrifugation with a 30 kDa-cutoff spin filter at 12 000 RCF for 7 min, washing with 200 µL of carbonate buffer, and with redispersion in carbonate buffer (300 µL, 100 mM, pH 9.3).

For SiO_2@(QD-Dex) assemblies, the initial SiO_2@QD assemblies (50 µL, ~0.35 nM) were mixed with API-Dex (12.5 µL, 28.5 mg/mL) for 1 h at 60 °C in the dark. SiO_2@(QD-Dex) were collected via centrifugation at 14 000 RCF for 10 min, washed with 50 µL of borate buffer three times, and finally dispersed in borate buffer (50 µL, pH 8.5, 50 mM). SiO_2@(QD-CM-Dex) were prepared using the same procedure, except with substitution of API-Dex by CM-Dex-API.

Cell Culture. Human SK-BR3 breast cancer cells (HTB-30, ATCC, Manassas, VA, USA) and human A549 lung cancer cells (ATCC CCL-185) were incubated in culture medium in a humidified incubator at 37 °C with 95% air/5% CO_2. The culture media was prepared by adding 10% v/v fetal bovine serum and 1X penicillin/streptomycin antibiotic and antimycotic (ThermoFisher) into McCoy's 5A medium (GE Healthcare, Chicago, IL). Cells were cultured in T25-flasks and the culture media was changed every 2–3 days to grow cells to confluency. Cells were then passaged into T75 flasks to confluency.

Human breast cancer MCF-7 (ATCC HTB-22) and MDA-MB-231 (ATCC HTB-26) cells were incubated in culture medium in a humidified incubator at 37 °C with 95% air/5% CO_2. The culture media was prepared by adding 10% v/v fetal bovine serum and 1X penicillin/streptomycin antibiotic and antimycotic into Dulbecco's Modified Eagle Medium (DMEM, Sigma Aldrich).

Cell Fixation. Paraformaldehyde-fixed cells were prepared by first trypsinizing the cells to detach them from the culture flask, collecting the culture media, and centrifuging in a 15 mL centrifuge tube at 55 RCF for 5 min to obtain a pellet of cells. The cells were redispersed in 2 mL of PBS buffer and then 2 mL of 4% w/v paraformaldehyde in PBS were added to the solution and left for 10 min. Fixed cells were pelleted via centrifugation at 55 RCF for 5 min. The cells were redispersed in PBS buffer and the final cell concentration was obtained using a Countess II automated cell counter (Invitrogen).

Ethanol-fixed cells were prepared by first chilling ethanol in a 15 mL centrifuge tube in dry ice for 30 min. Cultured SK-BR3 cells were trypsinized and collected as a pellet as described above. The pellet was then resuspended in 2 mL of 1X PBS buffer. Cell solution (2 mL) was then added to 8 mL of dry-ice-cold ethanol and incubated at –20 °C for 10 min. Fixed cells were centrifuged at

55 RCF for 5 min. The supernatant was discarded and the pellet was resuspended in 2 mL of 1X PBS buffer. The cell concentration of the final solution was obtained using the automated cell counter.

Unless otherwise noted, readers should assume that cells were fixed with paraformaldehyde rather than ethanol.

Smartphone FC Counting of Non-Specifically Labeled Cells (Figure S12). Ethanol-fixed SK-BR3 cells (200 µL) were pipetted into a 1.7 mL microcentrifuge tube and GSH-QDs (1 µL, ~3 µM) were added to the tube. The sample was mixed via pipette and then left in the dark for 20 min. The cells were centrifuged at 55 RCF for 5 min and washed with 200 µL of PBS buffer three times to remove unbound QDs. The cell pellet was resuspended in 200 µL of fresh PBS buffer.

Test samples of labeled cell suspensions with concentrations ranging from 9×10^2 to 2×10^5 cells/mL were prepared in 1.7 mL microcentrifuge tubes by dilution with PBS buffer. Each of the test samples, with a minimum volume of ~100 µL, was injected into the microfluidic chip. The test samples were pushed through the chip at a flow rate of 10 µL/min using a 1-mL syringe filled with deionized water and a syringe pump (KD Scientific, Holliston, MA). There was an air gap between the cell suspensions and the water. Cell counts and PL intensities were obtained by video analysis with a MATLAB algorithm (*vide infra*).

For validation, cell counts of the same test samples were also measured by pipetting an aliquot (10 µL) into a chamber slide for automated cell counting (Countess II Cell Counter, Invitrogen). Counts from the automated cell counter were used for validation of results with the smartphone FC. For expected cell counts that were below the lower limit of the cell counter (~10^4 cells/mL), validation of cell counts was instead obtained with a research-grade microscope. An aliquot (10 µL) of cell solution was pipetted into a cell counting chamber slide and the whole area was imaged under 4X objective, followed by particle analysis with ImageJ.

Formation of Tetrameric Antibody Complexes (TACs). TACs were prepared based on the manufacturer's protocol for the Do-It-Yourself Positive Selection Kit II (STEMCELL Technologies). The desired anti-target (*e.g.* HER2, MUC1, CD44, EpCAM) antibody (15 µg) was mixed with 100 µL of Component A and then 100 µL of Component B in order. The mixed sample was then incubated overnight at 37 °C and topped up to 1 mL with PBS buffer and stored at 4 °C for near-future use.

Labeling of Fixed SK-BR3 Cells with Dex-QDs and SiO_2@(QD-Dex) via TACs (Figure S6, Figure S7, Figure S14). Fixed SK-BR3 cells (50 µL) were pipetted into two separate 1.7 mL microcentrifuge tubes, followed by 5 µL of pre-formed TAC complexes with anti-HER2 antibody (81 nM). The solution was mixed and left in the dark for 15 min. Dex-QD635 (15 µL, ~0.5 µM in carbonate buffer) and SiO_2@(QD635-Dex) (10 µL, ~0.35 nM in borate buffer) were each added to one of the two tubes and left in the dark for another 15 min. The labeled cells were collected and washed three times with PBS buffer (50 µL) via centrifugation at 55 RCF for 5 min. The final cell pellet was redispersed in PBS buffer (50 µL). An analogous procedure was used for immunolabelling of other cell lines, but with different pre-formed TACs (Figure S7) or different SiO_2@(QDλ-Dex) assemblies (Figure S14).

Immunoconjugation of SiO_2@(QD-CM-Dex). SiO_2@(QD-CM-Dex) (5 µL, ~3 nM) were diluted into HEPES buffer (155 µL, 25 mM, pH ~7). Fresh solutions of 1-ethyl-3-(3-dimethylaminopropyl)-carbodiimide (EDC; 10 mg/mL, 10 µL) and *N*-hydroxysuccinimide (NHS; 10 mg/mL, 12 µL) were added to the SiO_2@(QD-CM-Dex), followed by 10 µL of anti-HER2 antibody (~1.1 µM). The solution was placed on a shaker for 1 h at room temperature in the dark. SiO_2@(QD-CM-Dex)-(anti-HER2) conjugates were then collected by centrifugation at 10 000 RCF for 5 min, followed by three washes with 200 µL of 0.5% w/v BSA in 1X PBS buffer. The conjugates were then redispersed in 20 µL of 0.05% w/v BSA in PBS buffer and stored at 4 °C for further use.

A procedure similar to the above was used for the conjugation of SiO_2@(QDλ-CM-Dex) with anti-MUC1 antibody (0.5 mg/mL), anti-CD44 antibody (0.5 mg/mL), and anti-ER antibody (1 mg/mL). Synthesized SiO_2@(QD-CM-Dex) (5 µL, ~3 nM) were diluted with HEPES buffer (155 µL). Next,

20 μL of 0.01% v/v Tween 20 was added to the 1.7 mL microcentrifuge tube to minimize non-specific binding of conjugates to the tube. The desired antibody (6 μL at 0.5 mg/mL for anti-MUC1, 6 μL at 0.5 mg/mL for anti-CD44, and 3 μL at 1.0 mg/mL for anti-ER) was added in this step to form SiO_2@(QD-CM-Dex)-antibody conjugates targeting different antigens. The solution was placed on a shaker for 1 h at room temperature in the dark. SiO_2@(QD-CM-Dex)-antibody conjugates were then collected by centrifugation at 10 000 RCF for 5 min, followed by three washes with 200 μL 0.5% w/v BSA in PBS buffer. The conjugates were redispersed in 20 μL 0.05% w/v BSA in PBS buffer and stored under 4 °C for further use.

Smartphone FC Counting SK-BR3 Cells Labeled with SiO_2@(QD635-Dex)-(anti-HER2 TAC) in the Absence of Background MDA-MB-231 Cells (Figure 3A). Paraformaldehyde-fixed SK-BR3 cells (300 μL, ~10^6 cells/mL) were pipetted into a 1.7 mL microcentrifuge tube and then mixed with 30 μL of DAPI solution (~2.9 μM in PBS buffer) and 30 μL of pre-formed TAC complex (81 nM). The mixture was left in the dark for 15 min. SiO_2@(QD635-Dex) (30 μL, ~0.3 nM) was then added to the solution and left in the dark for another 15 min. The cells were pelleted down via centrifugation at 55 RCF for 5 min, washed with Easy Sep buffer (300 μL), and redispersed in PBS buffer (300 μL).

An increasing number of SK-BR3 cells labeled with SiO_2@(QD635-Dex) was then pipetted from the stock solution into nine separate 1.7 mL microcentrifuge tubes and diluted up to 300 μL with 1X PBS buffer. For cell suspensions with a lower concentration (*i.e.* more than a 300-fold dilution), ~1 μL of stock solution was diluted with the desired amount of PBS buffer. The test samples (~100 μL for each run) were injected into the microfluidic chip and imaged with the smartphone-based flow cytometer. Smartphone videos (~3 min, ~30 μL of cell suspension) were analyzed with the MATLAB algorithm (*vide infra*).

Cell counts of each test sample were also obtained with an automated cell counter (Countess II Cell Counter, Invitrogen) for validation. For expected cell counts below the lower limit of the cell counter (~10^4 cells/mL), validation cell counts were obtained with a research-grade microscope. An aliquot (10 μL) of cell solution was pipetted into a chamber slide and the whole area was imaged under a 4X objective, followed by particle analysis with ImageJ.

Smartphone FC Counting SK-BR3 Cells Labeled with SiO_2@(QD635-Dex)-(anti-HER2 TAC) in the Presence of Background MDA-MB-231 Cells (Figure 3B). In two separate minicentrifuge tubes, SK-BR3 cells (300 µL, ~1.1 × 10^6 cells/mL) and MDA-MB-231 cells (300 µL, ~1.14 × 10^6 cells/mL) were redispersed in 100 µL of DAPI solution (~2.9 µM in PBS buffer) and left in the dark for 20 min. DAPI-stained cells were pelleted down at 55 RCF for 5 min and redispersed in 300 µL of Easy Sep buffer. Before preparation of the test samples, the DAPI-stained cells were filtered through a 40-µm cell strainer (STEMCELL Technologies).

A constant volume (50 µL, ~50k cells) of MDA-MB-231 cells were pipetted from a stock solution of ~1 × 10^6 cells/mL for each sample. An increasing number of SK-BR3 cells were pipetted into separate tubes: ~555, ~700, ~1320, ~1530, ~3750, and ~6320 fixed SK-BR3 cells were pipetted from a stock solution of ~234 500 cells/mL; ~8560, ~17 125, ~25 690, ~34 250, ~51 375, and ~60 000 fixed SK-BR3 were pipetted from a stock solution of ~342 500 cells/mL. Each of the test samples was diluted up to 300 µL with Easy Sep buffer.

Next, a spike of 0.5 µL of pre-formed TAC complex (81 nM) was added to each tube of fixed cells and left in the dark for 15 min, followed by 1 µL of SiO_2@(QD635-Dex) (~3.3 nM). The solution was left in the dark for another 15 min and then washed three times with 300 µL of Easy Sep buffer with centrifugation at 55 RCF for 5 min to remove excess SiO_2@(QD635-Dex). Cell pellets were redispersed in 600 µL of PBS buffer. Different test samples (~200 µL for each run) were imaged with the smartphone FC. Smartphone videos (~3 min, ~30 µL of cell suspension) were recorded and cell counts based on red (R) and blue (B) color channel intensities were obtained through the MATLAB algorithm. Counts were validated with the Countess II Cell Counter (Invitrogen) or a research-grade microscope (for counts < 10^4 cells/mL).

Selective Cell Labeling and Counting with SiO_2@(QD-CM-Dex)-Antibody Conjugates. The following paragraphs describe protocols for the various experiments:

Figure S8

An aliquot (50 µL, ~1.1 × 10^6 cells/mL) of fixed SK-BR3 cells (HER2+) and an aliquot (50 µL, ~1.14 × 10^6 cells/mL) of fixed MCF-7 cells (HER2–) were separately pelleted down and resuspended in 50 µL of Easy Sep buffer for immunolabeling with 3 µL of SiO_2@(QD-CM-Dex)-(anti-HER2) conjugates (~0.6 nM) for 1 h, followed by three washes with 50 µL of Easy Sep buffer at 55 RCF for 5 min, and redispersed in 50 µL of Easy Sep buffer. An aliquot (7.5 µL) of immunolabeled cell suspension was dropped on a microscope glass slide, a coverslip applied, and imaged under a research-grade microscope.

Figure 4B

Aliquots (50 µL, ~1.1 × 10^6 cells/mL) of fixed SK-BR3 cells were separately labeled with four colors (QD540, QD585, QD605, QD650) of SiO_2@(QD-CM-Dex)-(anti-HER2) conjugates (3 µL, ~0.6 nM) in Easy Sep buffer for 1 h in the dark, followed by three washes with 200 µL of Easy Sep buffer at 55 RCF for 5 min, and redispersed in 200 µL of PBS buffer. The cell suspensions were run through the smartphone FC and the videos were analyzed with the MATLAB algorithm.

Figure S9

Aliquots (20 µL, ~1 × 10^6 cells/mL) of fixed A549 cells (CD44+), fixed MDA-MB-231 cells (MUC1+), and fixed SK-BR3 cells (HER2+) were labeled separately with 1 µL of SiO_2@QD-antibody conjugate [SiO_2@(QD605-CM-Dex)-(anti-MUC1), SiO_2@(QD650-CM-Dex)-(anti-CD44), or SiO_2@(QD540-CM-Dex)-(anti-HER2)] in Easy Sep buffer for 1 h, followed by three washes with 50 µL of Easy Sep buffer at 55 RCF for 5 min, and redispersed in 20 µL of Easy Sep buffer. An aliquot (7.5 µL) of labeled cell solution was dropped on a microscope glass slide, a coverslip applied, and imaged under a research-grade microscope.

Figure S10

Aliquots (20 µL, ~1 × 10^6 cells/mL) of fixed MCF-7 cells (ER+), fixed SK-BR3 cells and MDA-MB-231 cells (ER–) were labeled separately with ~1 µL of SiO_2@(QD650-CM-Dex)-(anti-ER)

conjugates in Easy Sep buffer for 1h, followed by three washes with 50 µL of Easy Sep buffer at 55 RCF for 5 min, and redispersed in 20 µL of Easy Sep buffer. An aliquot (7.5 µL) of labeled cell solution was dropped on a microscope glass slide, a coverslip applied, and imaged under a research-grade microscope.

Figure 5 and Figure S18

Paraformaldehyde-fixed SK-BR3 (90 µL, ~132 000 cells) were pipetted from a stock solution of ~1 475 000 cells/mL for each sample. An increasing number of MDA-MB-231 cells were pipetted into separate tubes. Amounts of ~5100, ~25 500, ~51 000, ~102 000, ~153 000 fixed MDA-MB-231 cells were pipetted from a stock solution of ~1 020 000 cells/mL. Test samples of mixed cells were prepared, labeled with aliquots of SiO_2@(QD540-CM-Dex)-(anti-HER2) (3 µL ~0.6 nM) and SiO_2@(QD650-CM-Dex)-(anti-MUC1) (3 µL ~1 nM) for 1 h, washed with 200 µL of Easy Sep buffer three times, and resuspended in 600 µL of 1X PBS buffer.

The above samples were injected into the microfluidic chip, in sequence, from low cell concentration to high cell concentration, and imaged on the smartphone FC. The microfluidic chip was flushed with deionized water (~1 mL) three times between different samples. Smartphone videos (~2–3 min, ~20–30 µL of cell suspension) were recorded and cell counts in each classified group were obtained through the MATLAB algorithm. Expected total cell counts of each test sample mixture of SK-BR3 and MDA-MB-231 cells were obtained with an automated cell counter (Countess II Cell Counter, Invitrogen). Since the cell counter cannot distinguish between the two cell lines in the mixed test samples, the total cell counts of each test sample obtained from the cell counter were multiplied by the ratio of SK-BR3 and MDA-MB-231 cell stock solutions added. This calculation yielded the expected cell counts for the SK-BR3 and MDA-MB-231 cell lines in each test sample for validation of the results obtained with the smartphone FC.

Smartphone FC Classification of Breast Cancer Cell Lines with SiO_2@(QD-CM-Dex)-antibody conjugates (Figure 6 and Figure S11). Aliquots (50 µL) of paraformaldehyde-fixed SK-BR3 cells (~1.1×10^6 cells/mL), MDA-MB-231 cells (~1.14×10^6 cells/mL), and MCF-7 cells (~1.15×10^6 cells/mL) were pelleted down via centrifugation at 55 RCF for 5 min and resuspended in Easy Sep buffer (200 µL) in three separate microcentrifuge tubes. A combination of

SiO_2@(QD650-CM-Dex)-(anti-ER) (2 µL, ~1.0 nM), SiO_2@(QD585-CM-Dex)-(anti-MUC1) (3 µL, ~0.6 nM), and SiO_2@(QD540-CM-Dex)-(anti-HER2) (3 µL, ~0.6 nM) conjugates was then added to the microcentrifuge tubes and left in the dark on a shaker for 1 h. The cell solutions were pelleted down at 55 RCF for 5 min, washed with 200 µL of Easy Sep buffer three times, and resuspended in 200 µL of PBS buffer for each sample. A control sample with only the three types of SiO_2@(QD-CM-Dex)-antibody conjugates (no cells) was also prepared, washed, and pelleted down analogously as the test samples. For Figure 6, the cell suspensions and control sample were imaged with the smartphone FC and the videos were analyzed with the MATLAB algorithm. For Figure S11, an aliquot (7.5 µL) of labeled cell solution was dropped on a microscope glass slide, a coverslip applied, and imaged under a research-grade microscope.

Cell Tracking and Counting Algorithm. Smartphone videos (Samsung Galaxy S8, mp4 file format) of labeled cells were analyzed through a cell counting algorithm developed using the MATLAB R2019b computer vision toolbox. Each smartphone video was recorded under FHD mode at a frame rate of 30 frames per second (fps) with a resolution of 1920 × 1080 pixels. Quantification and classification of different cancer cells were done according to the color of SiO_2@QDs used for cell labeling and its RGB coordinates in the videos. The algorithm mainly included three main parts across four steps: detection (Step 1 and Step 2), tracking (Step 3), and classification (Step 4).

Step1 (Read Video):

Smartphone videos were imported and read frame by frame. Each frame was cropped to the relevant field of view (400 × 1920 pixels) and RGB color channels separated.

Step 2 (Foreground Detection):

Each video frame was converted to a binary image using the *vision.ForegroundDetector* function that compared a color or grayscale video frame to a background model to determine which pixels are part of the foreground and which are part of the background. Small holes inside the detected object, which is noted as a blob, were filled up with an *imfill* operator. Noise or small objects other than cells were removed based on the minimum blob area set by the user. This process was repeated for every frame in the video. A blob analysis system was used to compute the properties of each

visible object detected (*e.g.* blob areas, centroid, *etc.*) using the *vision.BlobAnalysis* function. The color coordinates (RGB values) of each object were extracted with the *regionprops* operator from the separated RGB channels.

Step 3 (Track Assignment):

An array of tracks was initialized to represent the moving objects in the video and to store the characteristics of each object. The predicted location of an object in the next frame was computed using the *Kalman filter* function. This function predicts the future location of an object and computes the Euclidean distance between the predicted centroid of the track and the centroid of the detection. Objects were then attributed to their corresponding tracks using Munkres' version of the Hungarian algorithm. This algorithm computes the cost of assigning the Nth detection to the Mth track and finds the assignment that minimizes the total cost (*i.e.* Euclidean distance between the prediction and the detection). In the end, the function returned a matrix containing the indices of assigned tracks and detections, as well as the indices and detections that were not assigned. For detected objects that were invisible for too long, the track was deleted from the track array. The maximum number of frames in which a detected object is permitted to be invisible was adjustable by the user (set as 8 in the experiments). After discarding the invalid tracks, all the reliable tracks were displayed in the video.

Step 4 (Classification and Export Results):

The data for all reliable tracks were exported to a comma-separated value (.csv) file with the following data: track number, blob pixel area, and mean RGB pixel intensities. For cells labeled via SiO_2@(QD-Dex)-(anti-target TAC) conjugates, a threshold for the ratio of Mean R and Mean B value was set to 1 to separate the red (QD labeled) and blue objects (DAPI stained only). For cells labeled with SiO_2@(QD-CM-Dex)-antibody conjugates, a linear SVM model was used to classify different colors (*vide infra*).

Area-based Correction for Cell Aggregates

A single-cell blob area threshold, set by the user, was used to discard any tracked objects that were much smaller than single cells. To mathematically correct for cell aggregates, the average area of each blob that was recorded during the object detection process (*vide supra*) was exported. A

histogram of these areas was obtained through the *histogram* operation in MATLAB and fitted with a non-parametric method via the *Distribution Fitter* app. From the histogram, an average area for a single labeled cell was determined (~102 pixels) and then used to calculate the area ratio between a detected object and the average single cell for all the experiments. The area ratio was rounded to the nearest integer using the *round* operation (*e.g.* if a detected object had an area of 220, it was identified as a double cell and counted as 2 cells). Any area ratio higher than 10 was discarded. Corrected cell counts were obtained by summing up the rounded area ratios for all detected objects.

The above process was also used for DAPI-stained MDA-MB-231 cells that were not labeled with SiO_2@(QD635-Dex) in the cell counting assay, and the single cell threshold was set to 77 pixels for blue objects. As DAPI is a nuclear stain, the threshold area of a single cell was expected to be smaller.

Support Vector Machine (SVM) Classification (Figure 5). A linear SVM model in MATLAB was used for classification of different cancer cell lines labeled with various colors of SiO_2@(QD-CM-Dex)-antibody conjugates. The two predictors were the Mean R/Mean B ratio value and the Mean G/Mean B ratio value obtained from the cell tracking algorithm.

To train the SVM model, SK-BR3 cells labeled with SiO_2@(QD540-CM-Dex)-anti-HER2 conjugates and MDA-MB-231 cells labeled with SiO_2@(QD650-CM-Dex)-anti-MUC1 conjugates were separately measured in the smartphone FC to generate two data sets with pre-determined "green" and "red" classes. A third "non-categorized" class was also used to train the SVM model and address the potential ambiguity between "red" and "green" near the origin of the R/B and G/B color ratio space. This training sample was fixed cells without any labeling with SiO_2@QD. The SVM model was trained in one epoch, with 5-fold cross-validation, using 2036 data points. Test samples were between 500–2200 data points. The confusion matrix for the SVM model training is shown in Figure S3.

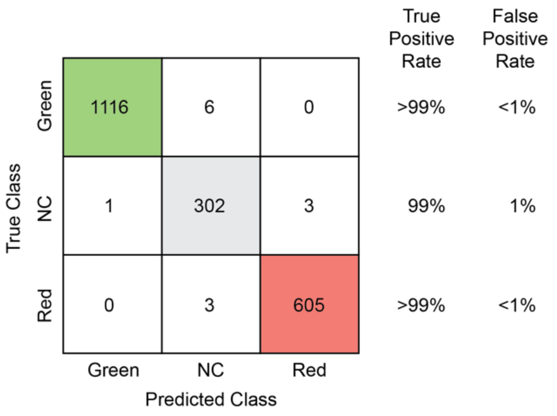

Figure S3. Confusion matrix for the SVM model training.

Experimentally, paraformaldehyde-fixed SK-BR3 cells (50 µL, ~ 1.1 × 10^6 cells/mL) and MDA-MB-231 cells (50 µL, 1.14 × 10^6 cells/mL) were separately incubated with aliquots of SiO_2@(QD540-CM-Dex)-(anti-HER2) (3 µL, ~0.6 nM) and SiO_2@(QD650-CM-Dex)-(anti-MUC1) (3 µL, ~1 nM) bioconjugates, respectively, in 1.7 mL microcentrifuge tubes for 1 h in the dark on a shaker at room temperature. Each set of labeled cells was pelleted down via centrifugation at 55 RCF for 5 min, washed with 200 µL of Easy Sep buffer three times, and redispersed in 200 µL of PBS buffer. These cell suspensions, plus the suspension of unlabeled cells, were injected into the microfluidic chip and imaged on the smartphone FC.

Color features extracted from video analysis of all three samples sets were normalized to span between values of 0 and 1 and were used as the training dataset for the SVM model. The normalization process made the two predictors for the test samples and training dataset fall into the same range and avoided any misclassification caused by the color feature differences (*e.g.* brightness) between the training and test samples. The data sets were imported to MATLAB and used with the *Classification Learner* app. The model accuracy was 99.4%. The SVM model was then exported and applied to the classification of test samples, ultimately providing cell counts for each class.

Supplementary Results and Discussion

Characterization of SiO_2@QD Assemblies. Figure S4 shows representative examples of optical spectra for SiO_2@QD assemblies. The NTA data is also summarized in Table S1. Note that we have previously reported more detailed characterization for these materials.[2]

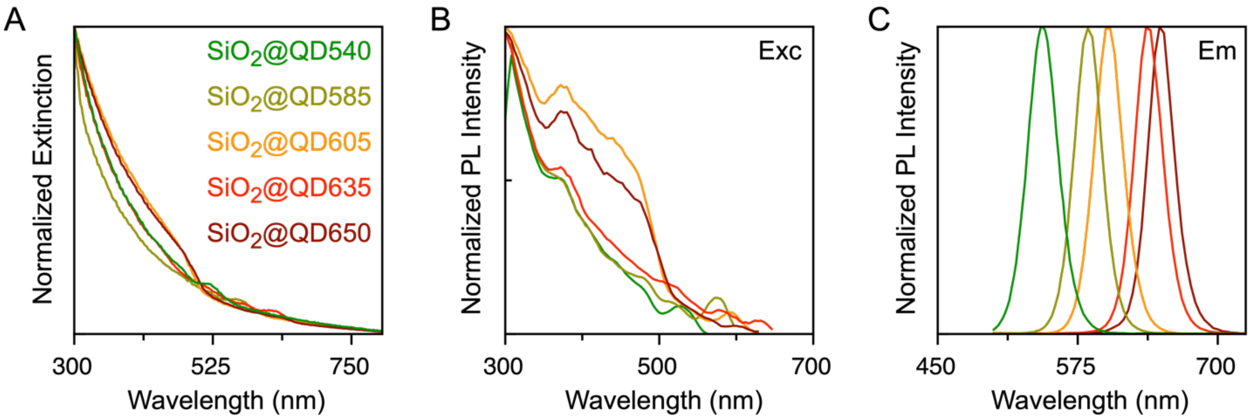

Figure S4. Examples of normalized **(A)** extinction (absorbance plus scattering), **(B)** PL excitation, and **(C)** PL emission spectra for five colors of SiO_2@QDλ assemblies. The extinction spectra show a combination of light absorption from the QDs and light scattering by the core SiO_2 nanoparticle.

Table S1. Mode hydrodynamic diameters (± standard deviation of size distribution) of SiO_2@(QD-Dex) and SiO_2@(QD-CM-Dex). The measured concentrations of the measured dilutions of the SiO_2@QD are also listed. These data were derived from scattering mode and fluorescence mode NTA.

Material	Scattering Mode NTA		Fluorescence Mode NTA	
	Diameter (nm)	Particles / mL	Diameter (nm)	Particles / mL
SiO_2@(QD650-CM-Dex)	88 ± 42	(1.6 ± 0.1) x 10^{12}	76 ± 41	(8.6 ± 0.4) x 10^{11}
SiO_2@(QD635-Dex)	92 ± 39	(4.1 ± 0.1) x 10^{12}	97 ± 41	(3.2 ± 0.1) x 10^{12}
SiO_2@(QD605-Dex)	117 ± 44	(1.8 ± 0.1) x 10^{12}	104 ± 52	(1.6 ± 0.4) x 10^{12}
SiO_2@(QD585-CM-Dex)	85 ± 31	(4.3 ± 0.2) x 10^{12}	83 ± 35	(3.8 ± 0.2) x 10^{12}
SiO_2@(QD540-CM-Dex)	83 ± 34	(1.6 ± 0.02) x 10^{12}	77 ± 30	(6.8 ± 0.3) x 10^{11}

Immunolabeling Strategies. Figure S5 shows schematics of the immunolabeling strategies used in this study. In the TAC strategy, an SiO_2@(QD-Dex) is bound to a cell through a complex of four antibodies: mouse anti-dextran IgG (blue in Figure S5A), mouse anti-target IgG (orange in Figure S5A with HER2 as the example of a target), and modified anti-mouse IgG antibodies (purple in Figure S5A). In the carbodiimide strategy, an SiO_2@(QD-CM-Dex) is bound to a cell through an anti-target IgG (orange in Figure S5B with HER2 as the example of a target) that is directly attached to the CM-Dextran via an amide bond.

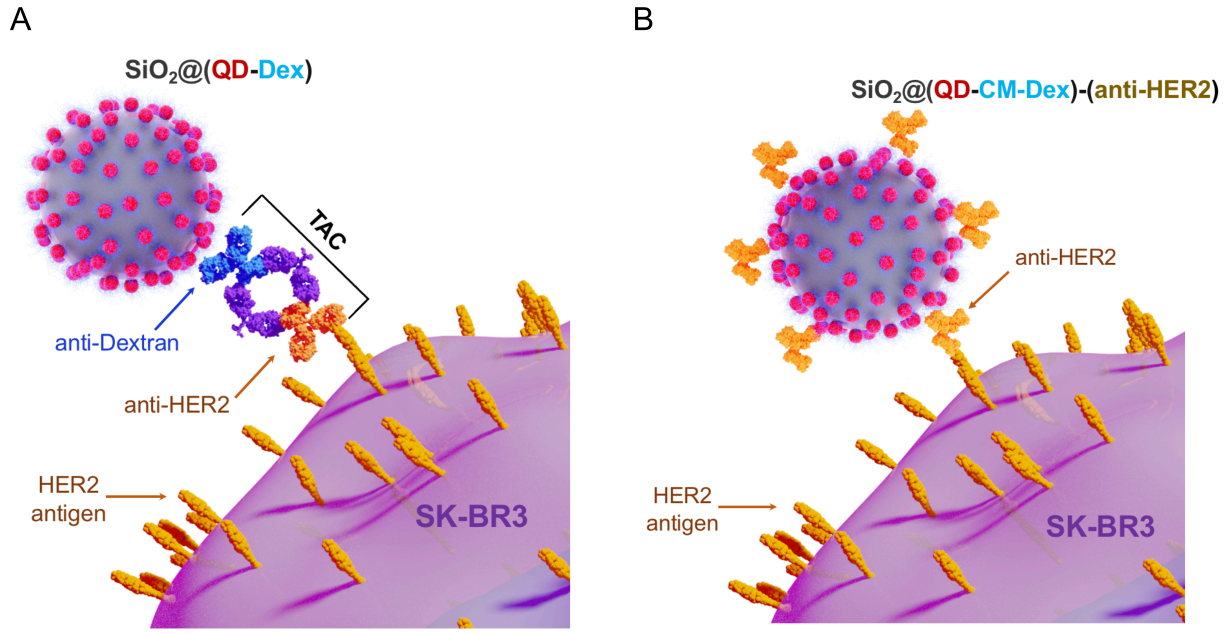

Figure S5. Illustrations of the **(A)** tetrameric antibody complex (TAC) and **(B)** carbodiimide strategies for immunolabeling cells with SiO_2@QD assemblies. HER2 is used as an example of a target antigen on the cell membrane.

Comparison of Immunolabeling with QDs versus SiO_2@QD. The microscope PL images in Figure S6 show that SiO_2@(QD-Dex)-(anti-HER2 TAC) provided much higher signal-to-background (SBR) and signal-to-noise (SNR) ratios than (QD-Dex)-(anti-HER2 TAC) for immunolabeling of fixed SK-BR3 cells.

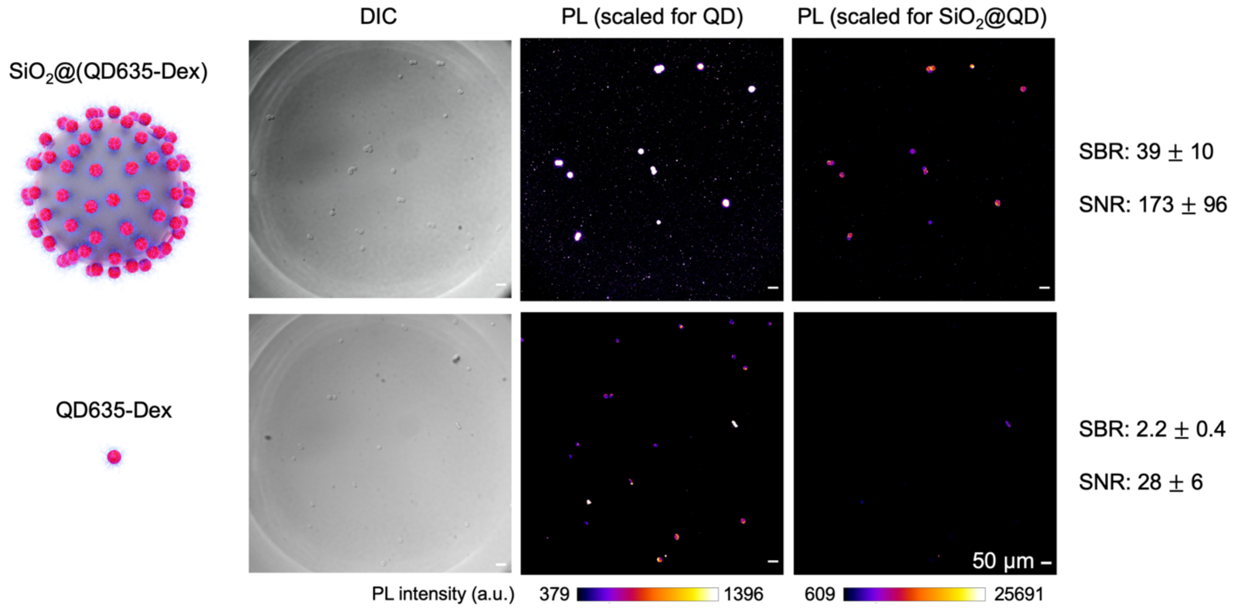

Figure S6. Comparison of anti-HER2 TAC-based immunolabeling of fixed SK-BR3 cells with individual QDs (QD-Dex) and supra-nanoparticle assemblies of SiO_2 NPs and QDs (SiO_2@(QD-Dex)). Microscope PL images (4X) are shown with the false-color scale optimized for both the individual QDs and the SiO_2@QDs, but are otherwise the same image. The scale bar in all images is 50 µm. The PL images for the QDs and the SiO_2@QDs were acquired at the same microscope settings.

TAC Immunolabeling of Various Antigens on Cancer Cells. The microscope PL images in Figure S7A show the potential to label the HER2, MUC1, and EpCAM antigens on different SK-BR3, MDA-MB-231 cells, and MCF-7 cells. SiO_2@(QD635-Dex) and TACs were used for these experiments. The TAC had mouse anti-dextran IgG and mouse anti-X IgG, where X is HER2, MUC1, or EpCAM. The expression levels, measured as SBRs and SNRs from PL intensities and shown in Figure S7B, and are consistent with expectations for these cell lines. This data allowed us to match the brightest colors SiO_2@QD with the antibodies targeting the genes/antigens that show the lowest expression levels, ideally optimizing signals across all antigens.

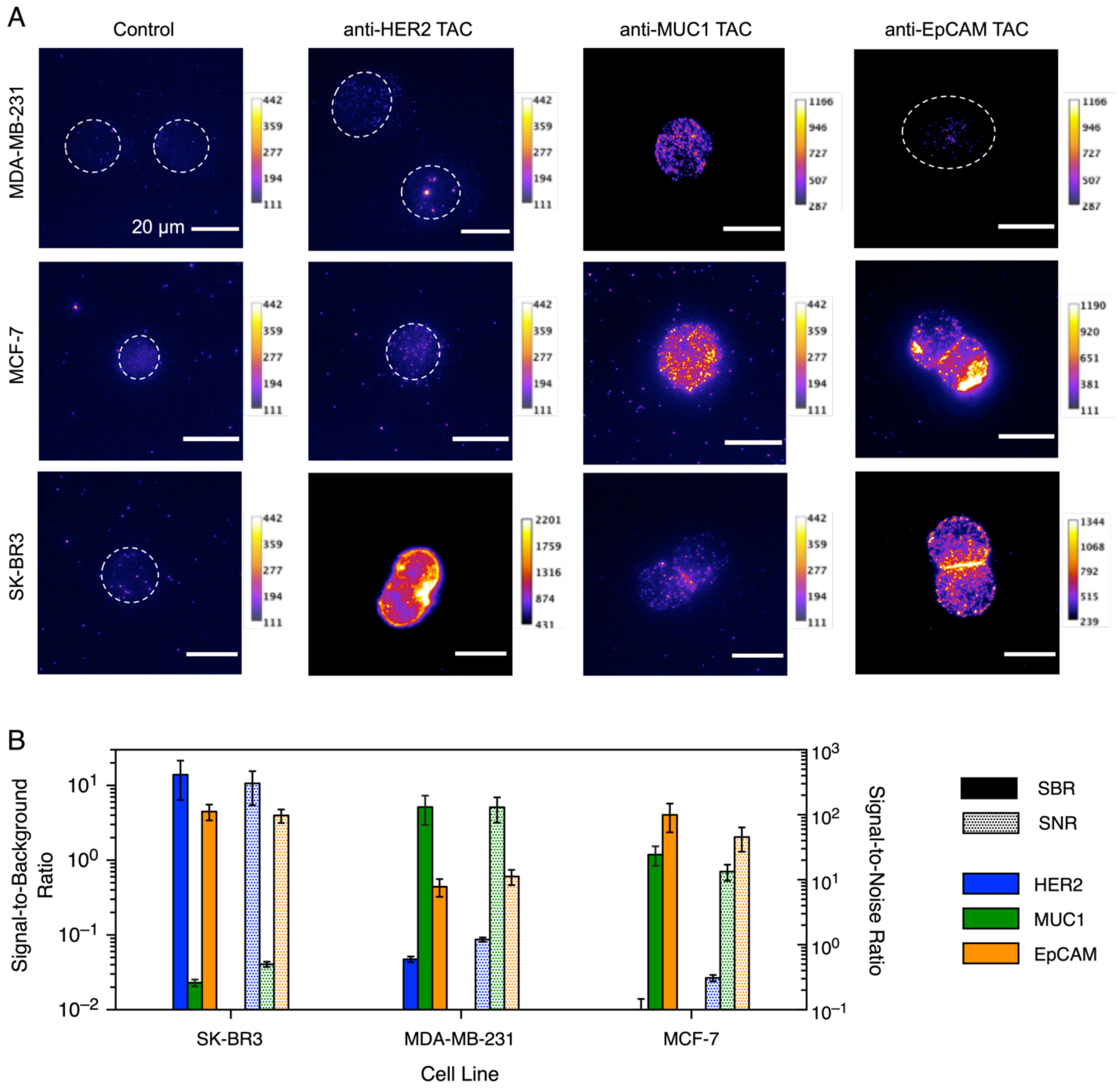

Figure S7. TAC-based immunolabeling of HER2, MUC1, and EpCAM on SK-BR3, MDA-MB-231, and MCF-7 cells. **(A)** Microscope PL intensity images (60X; false colored) of labeled cells. Control cells were incubated with SiO_2@(QD635-Dex) without TAC. Unlabeled cells are outlined with dashed circles. The scale bar is 20 μm. All images were acquired under the same microscope settings. **(B)** SBRs and SNRs for the immunolabeling of each antigen on each cell line. Error bars are one standard deviation. $N \geq 20$ cells were analyzed.

Verification of Selectivity of Carbodiimide-based Immunoconjugation. The microscope PL images in Figure S8 show that SiO_2@(QD-CM-Dex) efficiently labeled to HER2-positive SK-BR3 cells and negligibly labeled to HER2-negative MCF-7 cells when conjugated with anti-HER2 IgG via EDC/NHS. Significantly less labeling of SK-BR3 cells was observed when EDC was removed from the conjugation reaction with anti-HER2 IgG, with this residual binding to cells attributable to non-specific binding of anti-HER2 IgG to the SiO_2@(QD-CM-Dex). The SiO_2@(QD-CM-Dex) did not bind to SK-BR3 cells when unconjugated to anti-HER2 IgG.

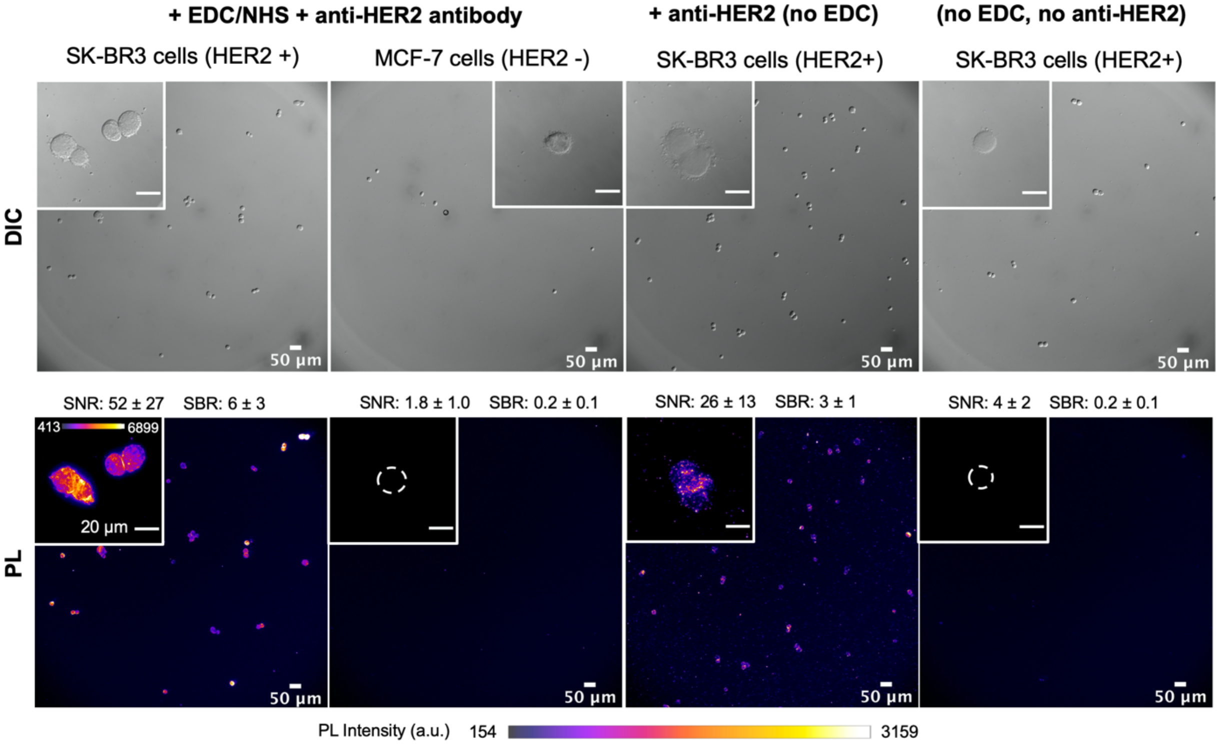

Figure S8. Immunolabeling of fixed SK-BR3 cells (HER2-positive) with carbodiimide-coupled SiO_2@(QD635-CM-Dex)-(anti-HER2) conjugates, with MCF-7 cells (HER2-negative) and SiO_2@(QD635-CM-Dex) as negative controls. Top row: DIC images. Bottom row: PL images. Scale bars are 50 µm for the main images (10X) and 20 µm for the insets (60X). All PL images were acquired under the same microscope settings. SBR and SNR values for the PL images were calculated from the 10X images.

Immunolabeling of Different Antigens with SiO_2@(QD-CM-Dex). Figure S9 shows microscope PL images and SBR and SNR values for MDA-MB-231, A549, and SK-BR3 cells after incubation with SiO_2@(QD605-CM-Dex)-(anti-MUC1), SiO_2@(QD650-CM-Dex)-(anti-CD44), and SiO_2@(QD540-CM-Dex)-(anti-HER2) conjugates. The expected dominant labeling of HER2 on SK-BR3 cells, CD44 on A549 cells, and MUC1 on MDA-MB-231 cells was observed. Similarly, Figure S10 shows microscope PL images that show the expected selective labeling of ER on MCF-7 cells when using SiO_2@(QD650-CM-Dex)-(anti-ER) conjugates.

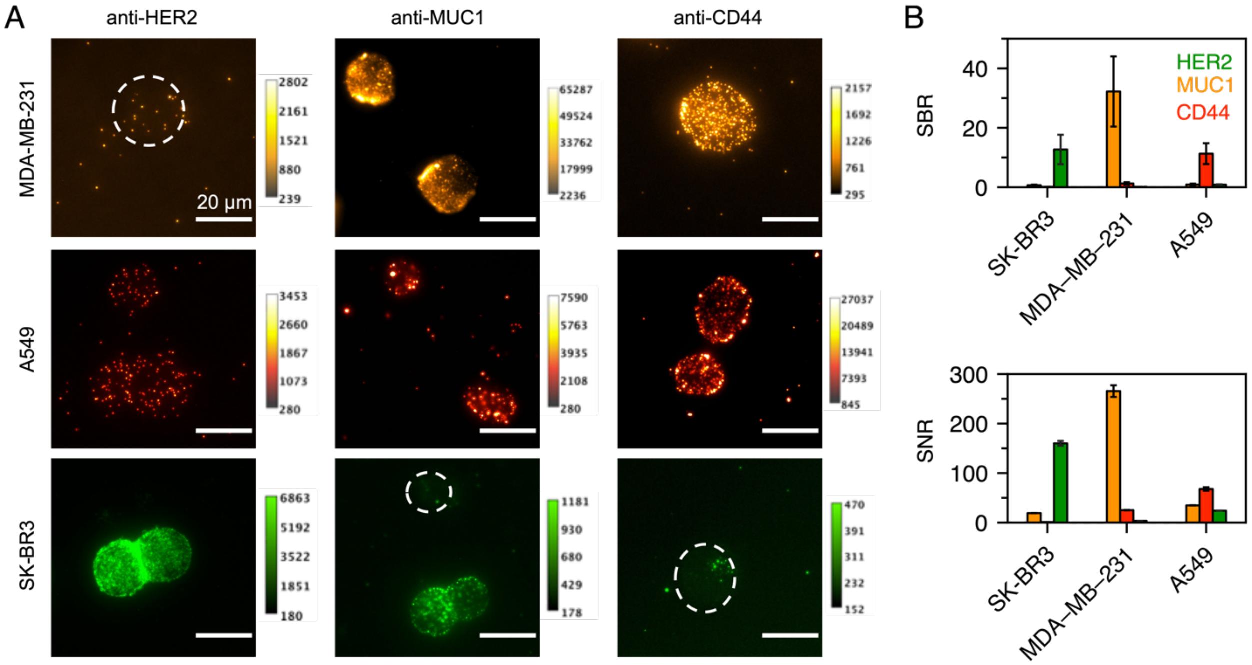

Figure S9. Immunolabeling of different antigens with SiO_2@(QD-CM-Dex)-(anti-target) conjugates across multiple cell lines. **(A)** Microscope PL images (60X). The images are false colored or pseudo-colored. Note the different color scales between images. All PL images in a row (*i.e.* common cell line) were acquired under the same microscope settings. **(B)** SBRs and SNRs for the immunolabeling of each antigen on each cell line. Error bars are one standard deviation. $N \geq 20$ cells were analyzed. All scale bars are 20 μm.

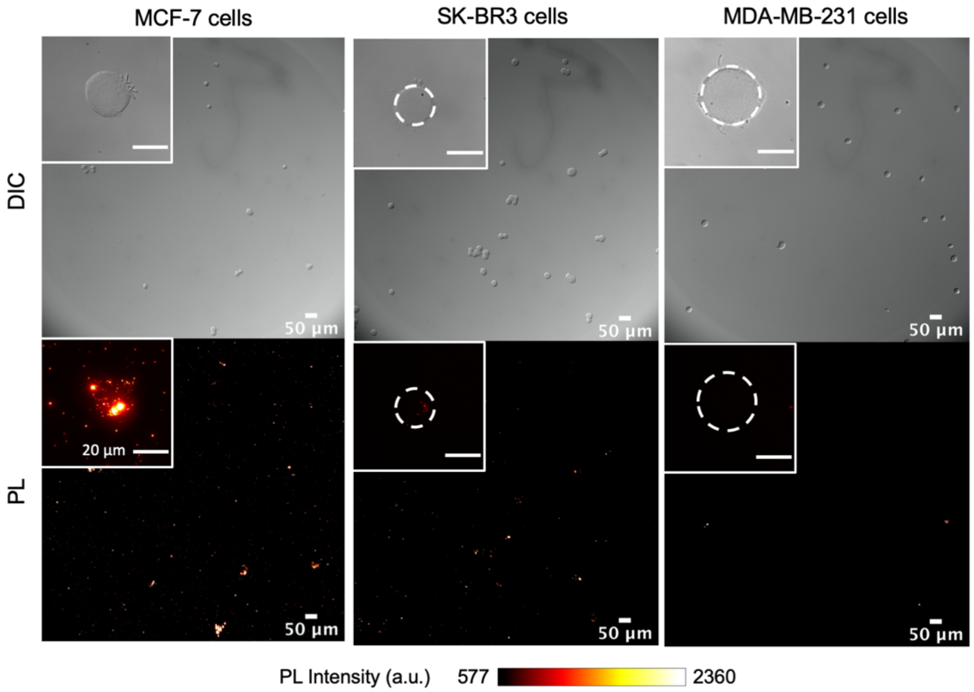

Figure S10. Microscope images showing selective immunolabeling of ER antigen on fixed MCF-7 cells with SiO_2@(QD650-CM-Dex)-(anti-ER) conjugates. SK-BR3 cells and MDA-MB-231 cells were used as negative controls. The PL images (10X main image, 50 µm scale bar; 60X inset image, 20 µm scale bar) are false colored. All PL images were acquired under the same microscope settings.

Figure S11 shows examples of PL images of fixed SK-BR3, MDA-MB-231, and MCF-7 cells after incubation with a combination of SiO_2@(QD650-CM-Dex)-(anti-ER), SiO_2@(QD585-CM-Dex)-(anti-MUC1), and SiO_2@(QD540-CM-Dex)-(anti-HER2). The SK-BR3 cells had a dominant signal from labeling of HER2 and minor signal from ER; the MDA-MB-231 cells showed only signal from labeling of MUC1; and the MCF-7 cells showed primary labeling of ER with secondary labeling of MUC1.

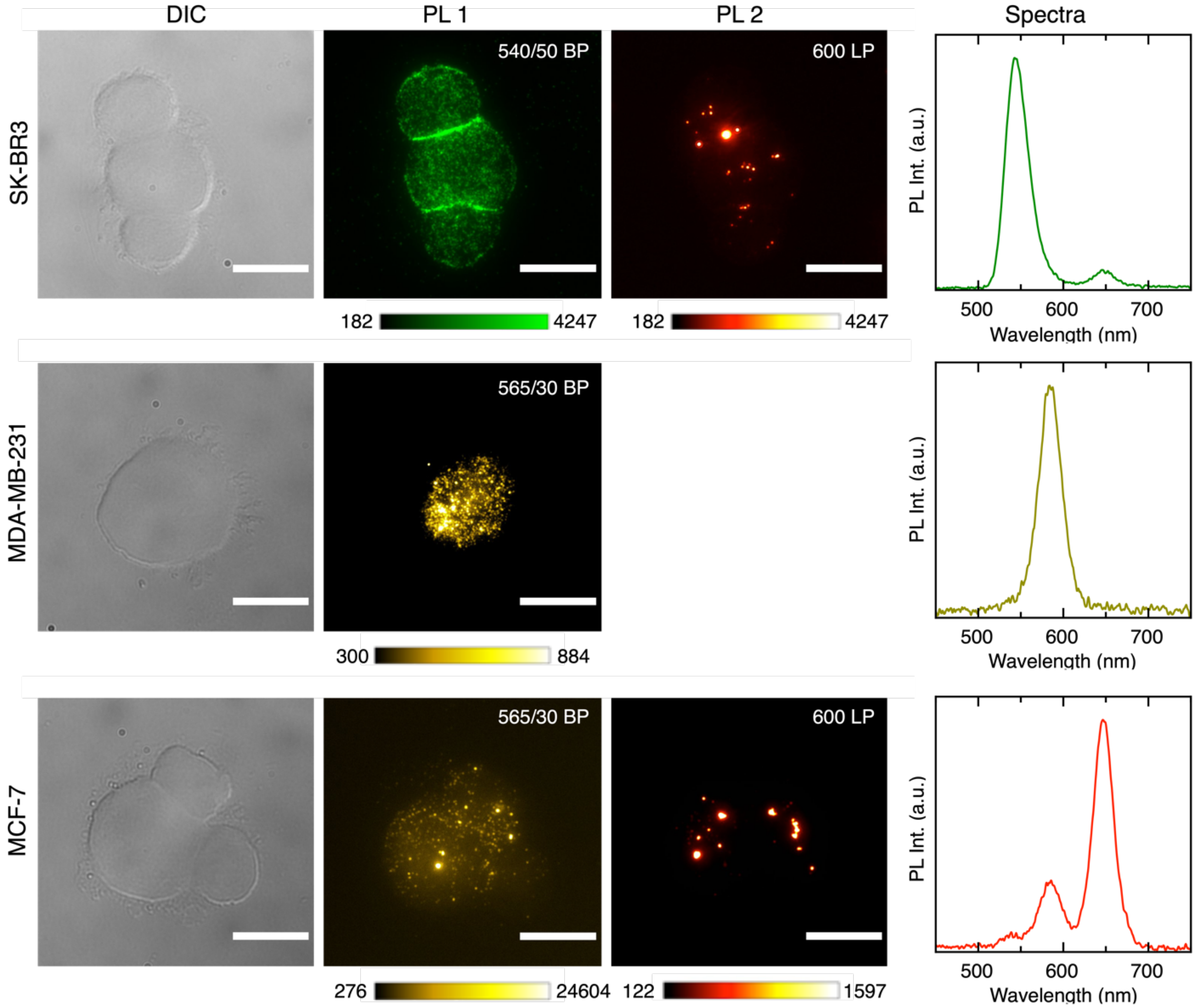

Figure S11. Examples of microscope images (60X, 20 μm scale bars) of fixed SK-BR3, MDA-MB-231, and MCF-7 cells after incubation with a combination of SiO_2@(QD650-CM-Dex)-(anti-ER), SiO_2@(QD585-CM-Dex)-(anti-MUC1), and SiO_2@(QD540-CM-Dex)-(anti-HER2). The emission filter used for each PL image is listed in the top-right corner (BP = band-pass, LP = long-pass). For MCF-7 cells, the shoulder near 525 nm is background and not QD540 PL.

Counting Non-Specifically Labeled Cells. In our experience, glutathione (GSH)-coated QDs show strong non-specific binding to ethanol-fixed SK-BR3 cells. Figure S12A shows images of smartphone FC video frames for various concentrations of GSH-QD635-labeled SK-BR3 cells. Plots of smartphone FC-obtained cell counts versus expected cell counts are shown in Figure S12B, with a 1:1 correlation between the two values (slope = 0.99). This data confirmed the ability of the smartphone FC device and analysis to produce accurate cell counts.

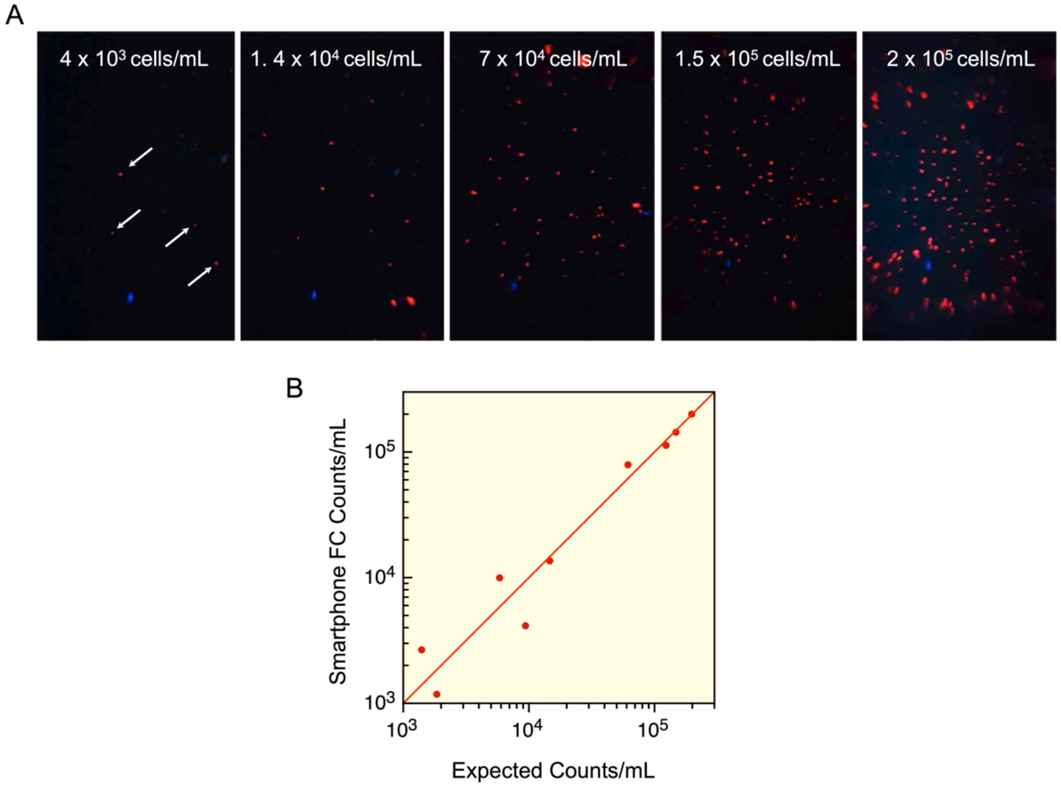

Figure S12. Counting of ethanol-fixed SK-BR3 cells non-specifically labeled with GSH-QD635. **(A)** Frames from smartphone FC videos recorded for different concentrations of suspended cells. For the most dilute suspension, the arrows indicate single cells. Frame image brightness has been digitally increased for display purposes. Video S2 is a 10 s clip from one of the smartphone FC videos. **(B)** Correlation plots of cell counts derived from the smartphone FC versus a commercial cell counter.

Counting a Single Cell Type. Figure S13 shows intensity profiles across selected cells for portions of the images in Figure 3.

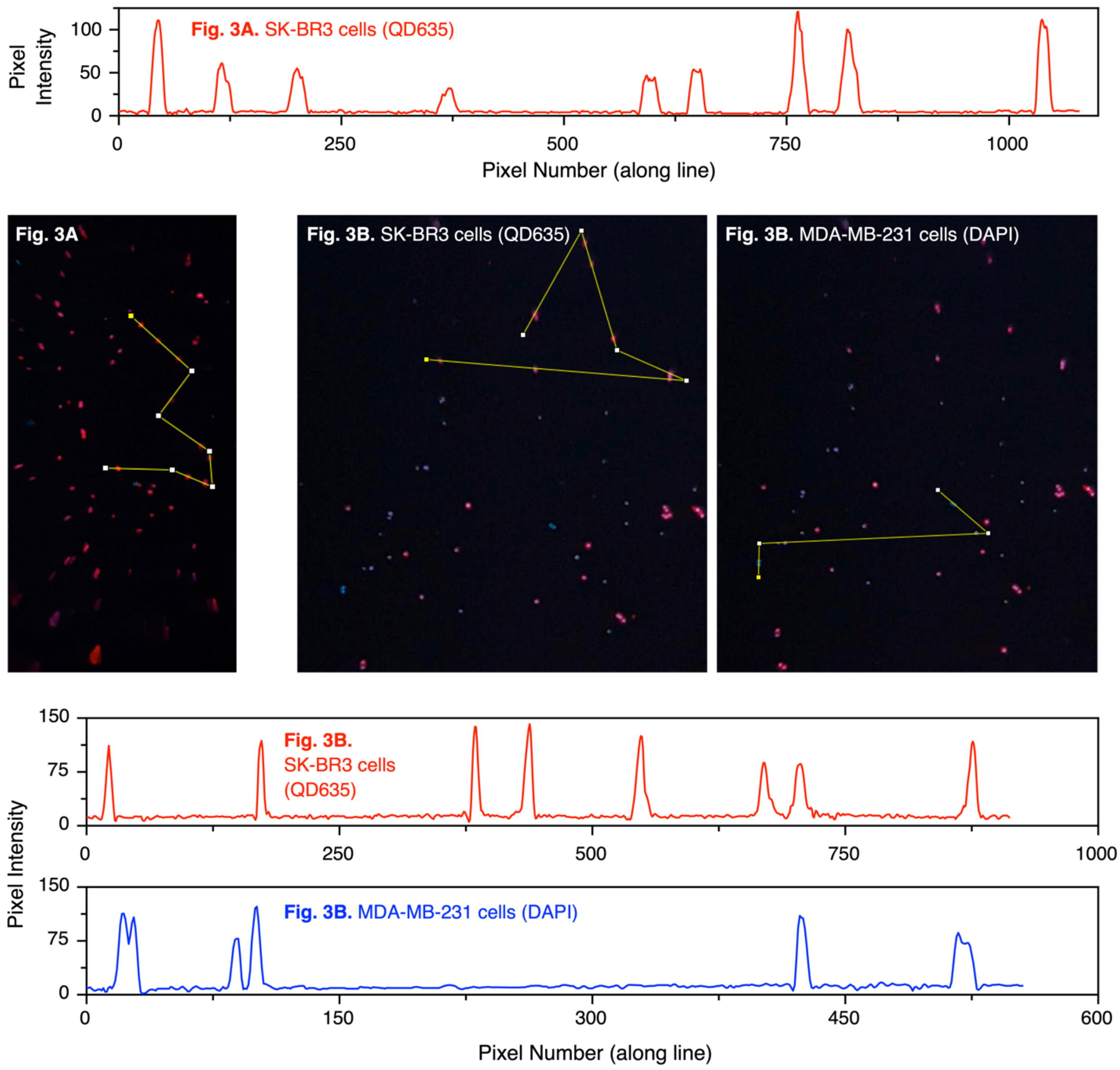

Figure S13. Images from Figure 3 with multi-segment lines drawn across some of the cells. The pixel intensity profiles along the drawn lines are shown below the images. The intensities are the mean of the RGB values. Note: The original images in Figure 3 have scale bars (~2.8 μm per pixel).

Multicolor Immunolabeling. Figure S14 shows PL images of fixed SK-BR3 cells separately labeled with SiO_2@(QD635-Dex), SiO_2@(QD605-Dex), SiO_2@(QD585-Dex) and SiO_2@(QD540-Dex), all with anti-HER2 TACs. The same samples were imaged on both a fluorescence microscope and with the smartphone FC device. Figure S15 shows plots of the G and R intensity values for individual SK-BR3 cells labeled with these four colors of SiO_2@(QDλ-CM-Dex)-(anti-HER2). Figure S16 shows intensity profiles across selected cells for portions of the images in Figure 4B.

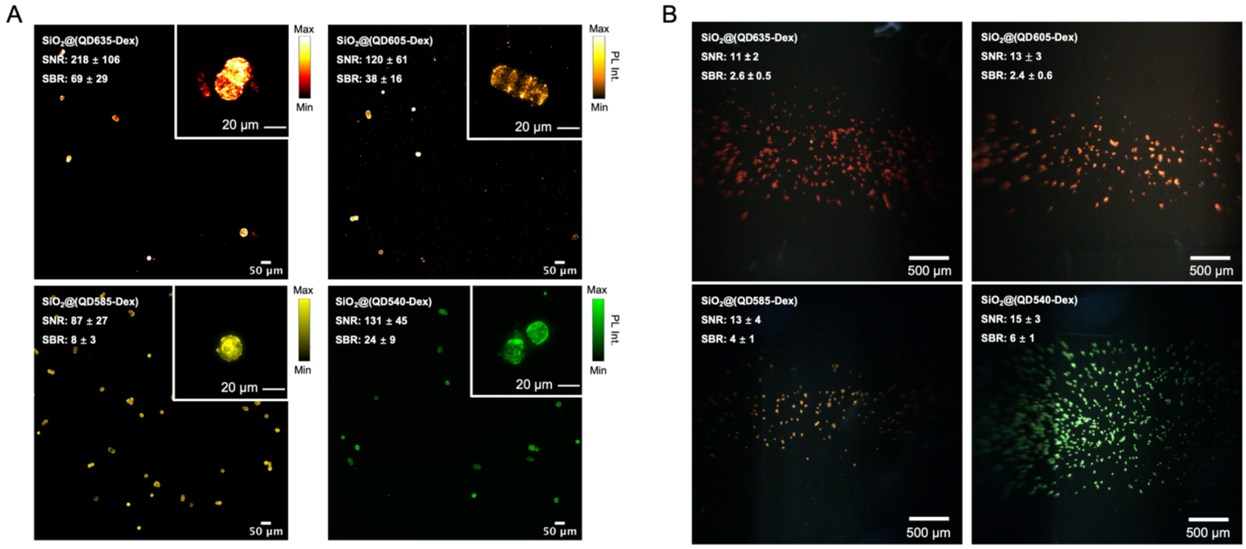

Figure S14. TAC-based immunolabeling of fixed SK-BR3 cells with various colors of SiO_2@(QDλ-Dex)-(anti-HER2). **(A)** Microscope PL images (10X) and calculated SBR and SNR ratios. The images are either false colored or pseudo-colored. **(B)** Smartphone RAW images of the same samples and calculated SBR and SNR ratios. All images were acquired with the same smartphone settings (ISO200, exposure time 1/30s). The flow direction was from right to left.

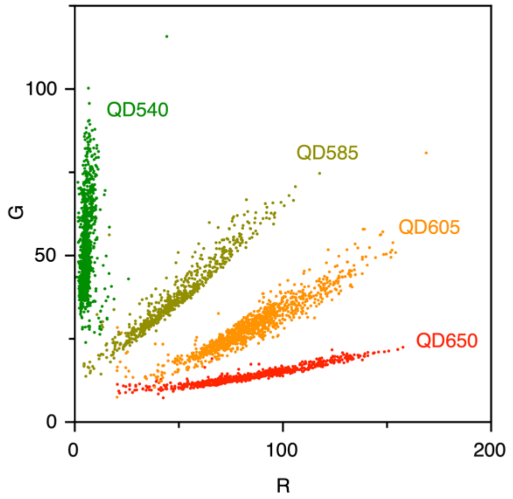

Figure S15. Data from Figure 4C in the main text, but without normalization to the B channel intensity.

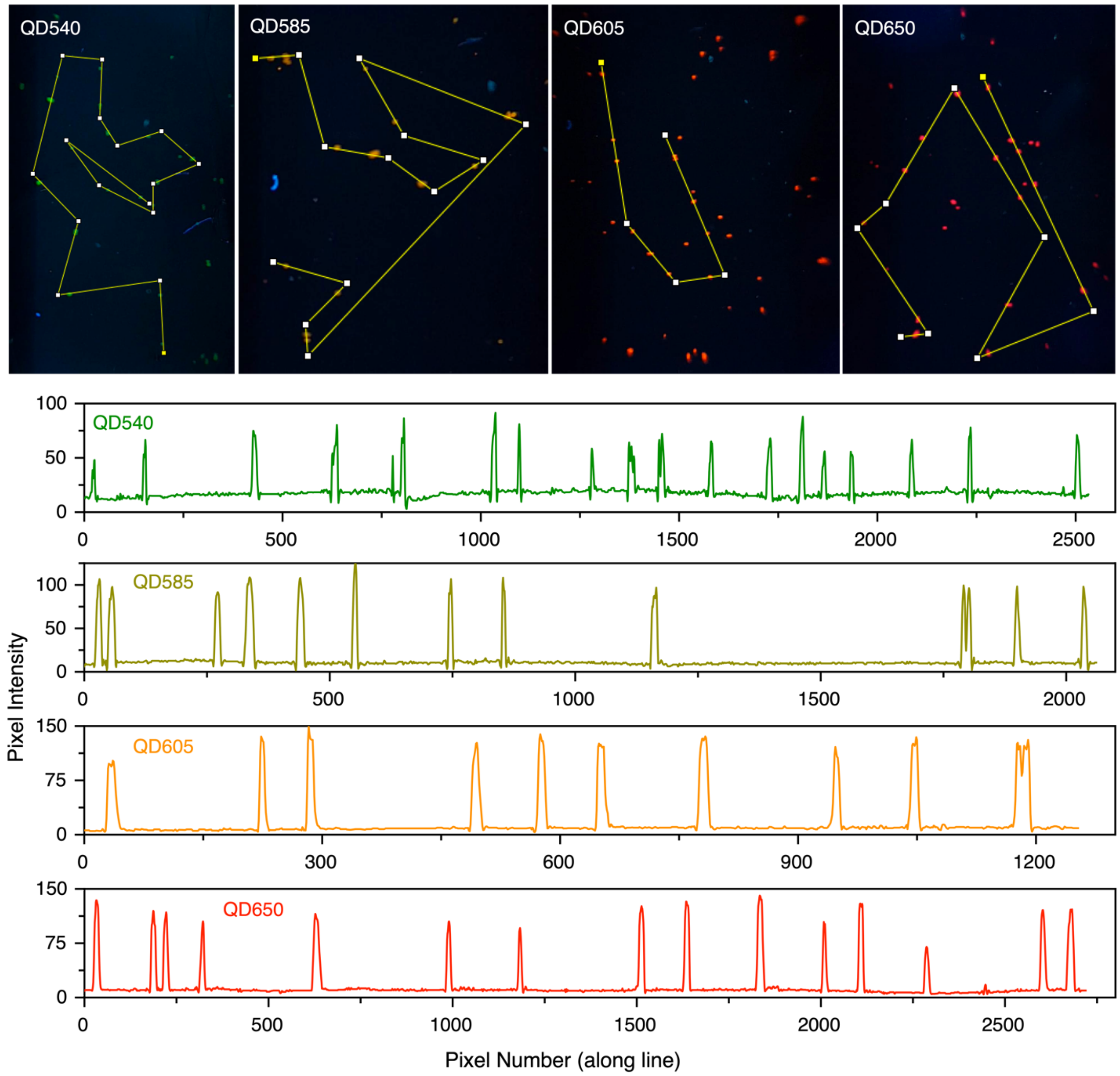

Figure S16. Images from Figure 4B with multi-segment lines drawn across some of the cells. The pixel intensity profiles along the drawn lines are shown below the images. The intensities are the mean of the RGB values. Note: The original images in Figure 4B have scale bars (~2.8 μm per pixel).

Additional Data for Two-Plex Cell Counting. Figure S17 shows intensity profiles across selected cells for portions of the image in Figure 5B.

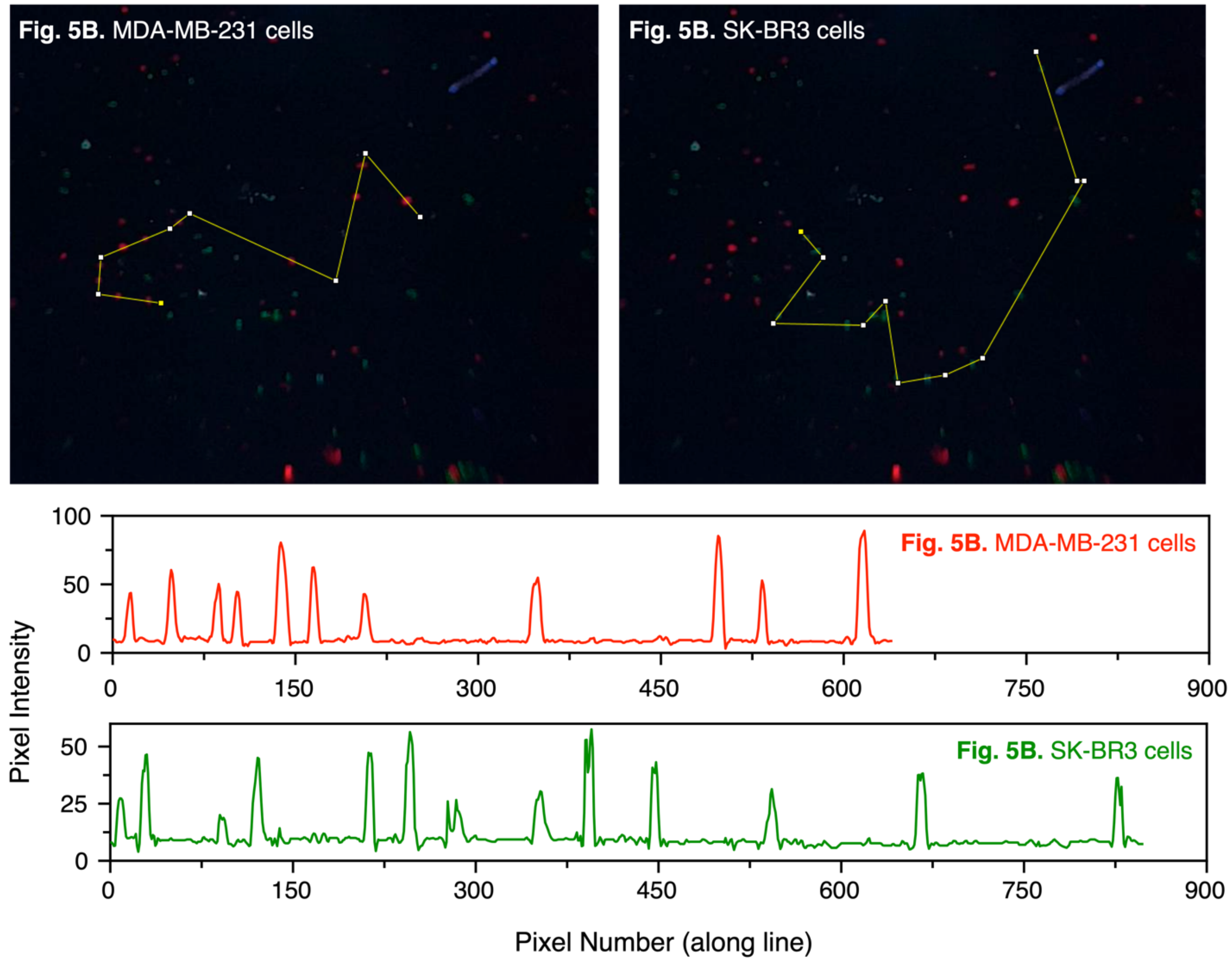

Figure S17. Image from Figure 5B with multi-segment lines drawn across some of the cells. The pixel intensity profiles along the drawn lines are shown below the images. The intensities are the mean of the RGB values. Note: The original image in Figure 5B has a scale bar (~2.8 μm per pixel).

Figure S18 shows additional data for two-plex counting of SK-BR3 cells and MDA-MB-231 cells with SiO_2@(QD540-CM-Dex)-(anti-HER2) and SiO_2@(QD650-CM-Dex)-(anti-MUC1). This data is not shown in Figure 5C, but is included in the summary in Figure 5D.

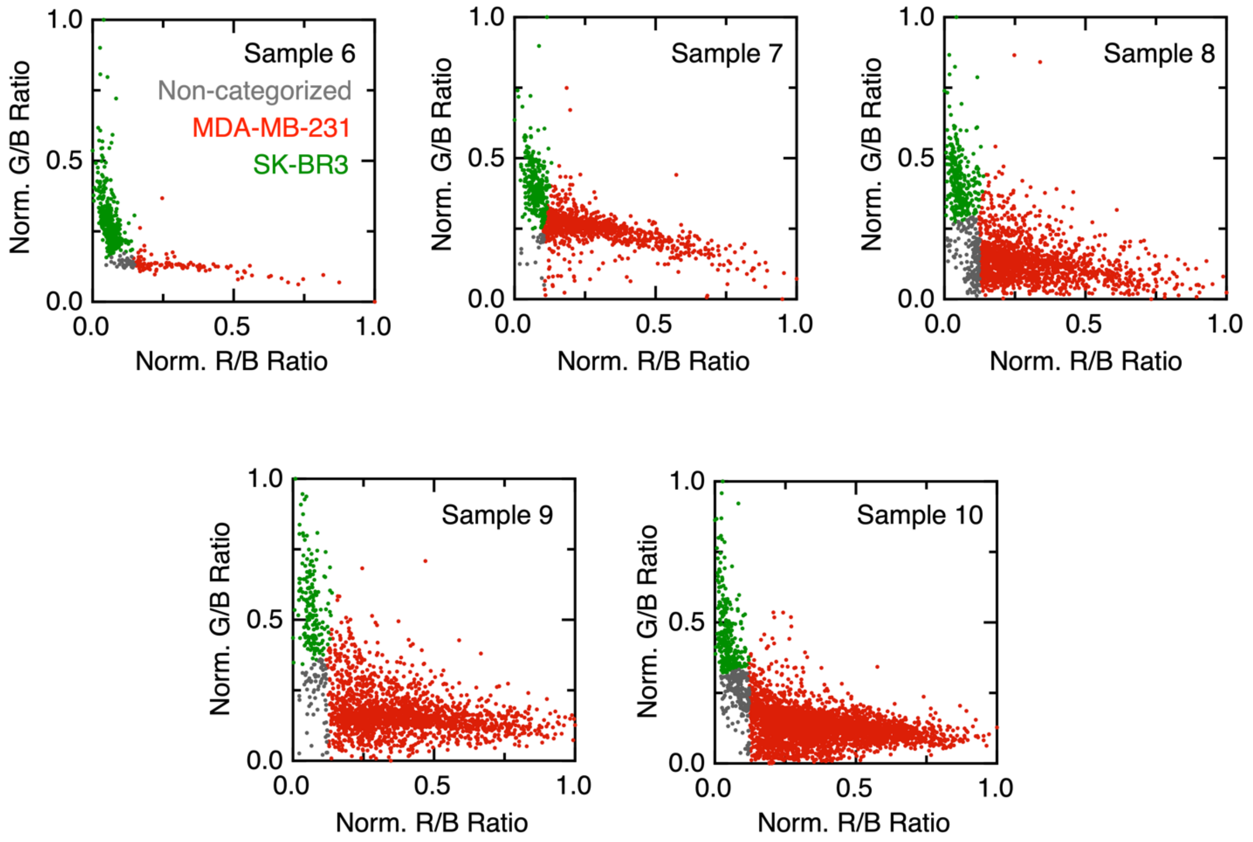

Figure S18. Examples of normalized G/B and R/B intensity ratios and cell classifications for five samples with an increasing number of MDA-MB-231 cells and an approximately constant number of SK-BR3 cells. This data is in addition to samples shown in Figure 5C. The data in this figure is included in Figure 5D.

Table S2. Maximum and minimum values for the measured R/B and G/B ratios for the normalized data in Figure 5 and Figure S18.

Sample	R/B Max	R/B Min	G/B Max	G/B Min
1 (Figure 5)	2.58	0.001	1.14	0.27
2	3.42	0.093	1.62	0.28
3	3.36	0.042	1.72	0.30
4	3.16	< 0.001	1.48	0.20
5	3.42	0.12	1.91	0.27
6 (Figure S18)	2.35	0.060	1.83	0.48
7	2.98	0.094	1.44	0.47
8	2.84	0.059	1.46	0.40
9	2.99	0.039	1.38	0.33
10	2.95	0.052	1.41	0.35

Potential Multiplexing Levels. Figure S19A shows hypothetical color trajectories in smartphone camera RGB color space for different SiO_2@QDλ, where λ would be expected to span from 460 nm to 650 nm. We anticipate that up to seven colors may be distinguishable when PL signals are sufficiently large to avoid the color space near the origin, where all trajectories converge. Given the smartphone camera filter transmission spectra in Figure 4A, the colors will most likely be limited to the RG and BG planes in the RGB color space. There is no QDλ that will simultaneously produce PL signal in both the blue and red channels.

Figure S19B shows hypothetical color trajectories in smartphone camera RGB color space for different SiO_2@QDλ_R/QDλ_G/QDλ_B. Here, the SiO_2@QDλ_R/QDλ_G/QDλ_B notation refers to SiO_2@QD prepared such that individual assemblies have a mixture of red-, green- and blue-emitting QDs. In this manner, it should be possible to access the full RGB color space, and perhaps up to eleven colors will be distinguishable (if measured at sufficient PL intensity).

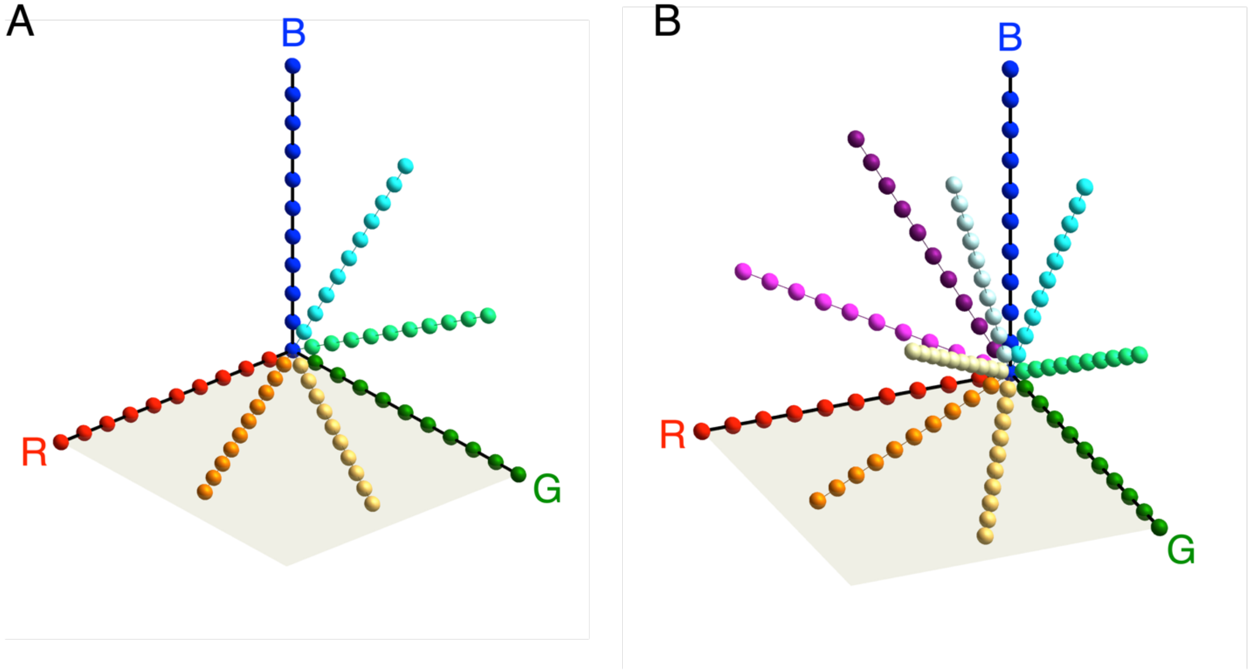

Figure S19. Hypothetical trajectories for filling the smartphone RGB color space with SiO_2@QD labels of different colors. The RG plane (used primarily in this study) is shaded. **(A)** Various SiO_2@QDλ will be able to span the RG and BG planes in the color space to achieve, for example, seven detectable label colors (when sufficiently far from the (0,0,0) origin). **(B)** We anticipate that various SiO_2@QDλ_R/QDλ_G/QDλ_B will be able to fill the color space, for up to eleven detectable label colors.

Supplemental References

(1) Susumu, K.; Field, L. D.; Oh, E.; Hunt, M.; Delehanty, J. B.; Palomo, V.; Dawson, P. E.; Huston, A. L.; Medintz, I. L. Purple-, Blue-, and Green-Emitting Multishell Alloyed Quantum Dots: Synthesis, Characterization, and Application for Ratiometric Extracellular PH Sensing. *Chem. Mater.* **2017**, *29*, 7330–7344.

(2) Darwish, G. H.; Asselin, J.; Tran, M. V; Gupta, R.; Kim, H.; Boudreau, D.; Algar, W. R. Fully Self-Assembled Silica Nanoparticle–Semiconductor Quantum Dot Supra-Nanoparticles and Immunoconjugates for Enhanced Cellular Imaging by Microscopy and Smartphone Camera. *ACS Appl. Mater. Interfaces* **2020**, *12*, 33530–33540.

(3) Stöber, W.; Fink, A.; Bohn, E. Controlled Growth of Monodisperse Silica Spheres in the Micron Size Range. *J. Colloid Interface Sci.* **1968**, *26*, 62–69.

(4) Tran, M. V; Susumu, K.; Medintz, I. L.; Algar, W. R. Supraparticle Assemblies of Magnetic Nanoparticles and Quantum Dots for Selective Cell Isolation and Counting on a Smartphone-Based Imaging Platform. *Anal. Chem.* **2019**, *91*, 11963–11971.
